# Antimicrobial Resistance Profiles of *Pseudomonas aeruginosa* in the Arabian Gulf Region Over a 12-Year Period (2010–2021)

**DOI:** 10.1007/s44197-024-00191-y

**Published:** 2024-06-10

**Authors:** A. Alatoom, M. Alattas, B. Alraddadi, C. Ayoub Moubareck, A. Hassanien, W. Jamal, A. Kurdi, N. Mohamed, A. Senok, A. M. Somily, H. Ziglam

**Affiliations:** 1 National Reference Laboratory, Abu Dhabi, UAE; 2 Present Address: Department of Pathology and Laboratory Medicine, Sheikh Shakhbout Medical City, Abu Dhabi, UAE; 3 King Faisal Specialist Hospital and Research Center, Jeddah, Saudi Arabia; 4 Alfaisal University, Riyadh, Saudi Arabia; 5 College of Natural and Health Sciences, Zayed University, Dubai, UAE; 6 Pfizer, Jeddah, Saudi Arabia; 7 Department of Microbiology, College of Medicine, Kuwait University, Jabriya, Kuwait; 8 Pfizer, Dubai, UAE; 9 Present Address: Hikma Pharmaceuticals, Amman, Jordan; 10 Pfizer Inc., New York, NY USA; 11 College of Medicine, Mohammed Bin Rashid University of Medicine and Health Sciences, Dubai, UAE; 12 Department of Pathology and Laboratory Medicine, College of Medicine, King Saud University Medical City, Riyadh, Saudi Arabia; 13 Department of Infectious Diseases, Hamad Medical Corporation, Doha, Qatar

**Keywords:** *Pseudomonas aeruginosa*, Arabian Gulf region, Antimicrobial resistance, Resistance mechanisms, Resistance phenotypes

## Abstract

**Objectives:**

To evaluate literature from a 12-year period (2010–2021) on the antimicrobial resistance profile of *Pseudomonas aeruginosa* from the Arabian Gulf countries (Bahrain, Kuwait, Oman, Qatar, Saudi Arabia, and the United Arab Emirates).

**Methods:**

An electronic literature search was conducted for articles on antimicrobial resistance in *P. aeruginosa* and associated phenotypes, covering the period of 1st January 2010 to 1st December 2021.

**Results:**

Antimicrobial resistance in the Arabian Gulf was highest to meropenem (10.3–45.7%) and lowest to colistin (0.0–0.8%), among the agents tested. Annual data showed that ceftazidime resistance (Kuwait), piperacillin-tazobactam non-susceptibility (Qatar), and aztreonam, imipenem, and meropenem resistance (Saudi Arabia) increased by 12–17%. Multiple mechanisms of carbapenem resistance were identified and multiple clones were detected, including high-risk clones such as ST235. The most common carbapenemases detected were the VIM-type metallo-β-lactamases.

**Conclusions:**

Among *P. aeruginosa* in the Arabian Gulf countries, resistance to meropenem was higher than to the other agents tested, and meropenem resistance increased in Saudi Arabia during the study period. Resistance to colistin, a classic antibiotic used to treat *Pseudomonas* spp. infections, remained low. The VIM-type β-lactamase genes were dominant. We recommend local and regional antimicrobial resistance surveillance programs to detect the emergence of resistance genes and to monitor antimicrobial resistance trends in *P. aeruginosa*.

**Supplementary Information:**

The online version contains supplementary material available at 10.1007/s44197-024-00191-y.

## Introduction

Globally, *Pseudomonas aeruginosa* is a leading cause of nosocomial infections [1], and in 2019, was estimated to have caused more than 500,000 deaths across 11 infection types, and more than 250,000 deaths associated with antimicrobial resistance [2, 3]. *P. aeruginosa* is an opportunistic pathogen exhibiting multiple mechanisms of resistance to multiple classes of antibiotics, including efflux pumps, enzymatic degradation, and target site modification [4]. *P. aeruginosa* is intrinsically-resistant to many antimicrobials, including members of the cephalosporins and fluoroquinolones [4]. As a result, multidrug-resistant (MDR; defined as non-susceptible to ≥1 agent in ≥3 antimicrobial classes [5]) *P. aeruginosa* has been listed as a ‘serious threat’ [6] and carbapenem-resistant (CR) *P. aeruginosa* has been designated a ‘Priority 1/critical’ pathogen [7]. Some CR strains produce carbapenemases, with VIM being the most common carbapenemase in *P. aeruginosa*, globally [8, 9].

*P. aeruginosa* with difficult-to-treat resistance (DTR-*P. aeruginosa*; defined as non-susceptible to piperacillin-tazobactam, ceftazidime, cefepime, aztreonam, meropenem, imipenem-cilastatin, ciprofloxacin and levofloxacin [10]) has also been highlighted in treatment guidance from the Infectious Diseases Society of America (IDSA) [11]. Treatment choices for antimicrobial-resistant *P. aeruginosa* should be dependent on local antimicrobial susceptibility patterns; however, the polymyxins, carbapenems, and aminoglycosides are available antipseudomonal options [4, 11]. For infections caused by DTR-*P. aeruginosa*, novel agents such as ceftolozane-tazobactam or ceftazidime-avibactam may be prescribed [4, 11].

The Arabian Gulf region is burdened by the same challenges of treating antimicrobial-resistant *P. aeruginosa* infections as other geographical regions; however, fewer data from this region are available compared with others. Therefore, this review aims to present currently available literature describing the antimicrobial resistance profile of *P. aeruginosa* and to identify the gaps in data from selected countries of the Arabian Gulf region: Bahrain, Kuwait, Oman, Qatar, Saudi Arabia, and the United Arab Emirates (UAE).

## Material and Methods

### Search Strategy and Selection Criteria

This review evaluated relevant studies addressing isolates of *P. aeruginosa*, published between 1st January 2010 and 1st December 2021 on the PubMed (Medline^®^), PubMed Central and Embase^®^ databases. The search terms included keywords from MeSH and Emtree thesaurus. The primary search term was “*Pseudomonas aeruginosa*”, including synonyms or variations thereof. In addition, synonyms for the Arabian Gulf region were included, and the Arabian Gulf country names (Bahrain, Kuwait, Oman, Qatar, Saudi Arabia, and the UAE). The mid-search terms relating to the review topics were “resistance” and “infections”, including any synonyms or variations. A free text search with those terms in the title and abstract fields was also performed. Examples of the synonyms or variations for the search terms are shown in Supplementary Table 1.

Accepted publication types included journal articles, systematic reviews, meta-analyses, clinical studies, randomised clinical trials, and global/regional/national reports. Case reports, letters, editorials, notes, conference abstracts, and non-English publications were excluded, as were study abstracts with no mention of *P. aeruginosa* or <10 isolates tested, unclear methodology, or environmental samples. Extracted study information from each article are presented in Table 1 (study findings) and Supplementary Table 2 (study methodologies). Study data are presented in Table 1, 2, Figs. 2, 3, and Supplementary Tables 3, 4.

**Table 1:** Summary table of study findings on antimicrobial resistance in *P. aeruginosa* from the Arabian Gulf countries (2010–2021)

Study reference, country	Study aims	*P. aeruginosa* isolates (*N*)	Study findings
Joji et al. [16], Bahrain	To study the presence of the metallo‑beta‑lactamase *(MbL)* genes of *VIM* family and *NDM‑1* in carbapenem‑resistant *P. aeruginosa* strains	50 (CR)	Among 40 IPM‑R strains, 21 (52%) were carrying MBL genes. Nineteen (47.5%) strains were positive for the *VIM* gene; one (2.5%) strain was carrying the *NDM‑1* gene, while one strain was carrying both *VIM* and *NDM‑1*. No IPM-sensitive strains carried *VIM* or *NDM‑1* genes. This is the first study to report the presence of the *VIM* family gene and *NDM‑1* genes in IPM-R *P. aeruginosa* isolates in the Kingdom of Bahrain. The study also confirms the multiple drug resistance by the MBL strains
Al Rashed et al. [17], Bahrain	To study efflux pump‑mediated fluoroquinolone resistance among *P. aeruginosa* isolates using phenotypic (E‑test and agar dilution) and genotypic (real‑time‑polymerase chain reaction) methods	50 (CIP‑R)	Four isolates showed reduction in CIP MIC after addition of carbonyl cyanide 3‑chlorophenylhydrazone. These four isolates showed upregulation of expression of at least one of the four genes by RT‑PCR. The mean gene expression of *MexB*, *MexD*, *MexF,* and *MexY* increased by 1.6, 4.65, 3.4, and 3.68‑fold, respectively. The results demonstrate the presence and type of efflux pump overexpression
Alfouzan et al. [18], Kuwait	To investigate the antimicrobial susceptibility patterns of selected strains (those with MDR profile) of *E. coli*, *K. pneumoniae*, and *P. aeruginosa*, which were isolated from various clinical specimens	48	*P. aeruginosa* isolates presented susceptibility rates of 97.9% to CST; followed by 47.9% to AMK; 39.6% to CZA; and 33.3% to C/T. Only one isolate was found to be CST-R and PDR
Alhubail et al. [19], Kuwait	To determine the microbiological profile of diabetic foot ulcers in patients attending Dasman Diabetes Institute clinics in Kuwait and to analyze the distribution of microbial isolates according to wound grade, sex, age and diabetes control	102	*P. aeruginosa* was predominantly found in ulcers with infection and ischemia as reflected by the number of patients in each of the diabetic foot ulcer grades. Antimicrobial susceptibility was highest to MEM (88.4%), AMK (87.0%), IPM (85%), and GEN (80.0%); and was lowest to CAZ (65.7%)
Al Rahmany et al. [20], Oman	To identify the prevalence of resistant pathogens and their susceptibility pattern in Northern Oman	2362 (2 ESBL-producers)	Among all 15,733 isolates. 22.2% were *P. aeruginosa*. ESBL enzyme production was rare in *P. aeruginosa* (2/2362 [0.1%] cases). The highest susceptibility of *P. aeruginosa* was to CST (100%), followed by TZP (93%) and CAZ (90%)
Balkhair et al. [21], Oman	To address the monthly cumulative frequencies and prevalence rates of hospital-acquired multidrug-resistant organisms during the year of 2012. Also, to describe the distribution of hospital-acquired multidrug-resistant organisms by site of infection and types of bacteria	27	Among all 329 isolated MDR organisms, the percentage of *P. aeruginosa* was 8.1%
Balkhair et al. [22], Oman	To examine the burden of carbapenem colistin resistance in *Klebsiella pneumoniae*, *Pseudomonas aeruginosa*, and *Acinetobacter baumannii* blood isolates, describe trends in carbapenem resistance in blood isolates over a 10-year period, and investigate 30-day all-cause mortality in patients with bacteraemia caused by CR isolates in Oman	231 (41 CR)	Among all 775 bacteremia isolates, 30% were *P. aeruginosa* (69% of *P. aeruginosa* were healthcare-associated and 31% were community-acquired). *P. aeruginosa* caused 18.1% of all CR bacteremia (88% of which was healthcare-associated and 12% community-associated). The overall rate of carbapenem resistance was 18.6% (41/221); the yearly rate increased from 20% in 2007 to 25% in 2016. All three patients with *P. aeruginosa* bacteremia that was carbapenem- and CST-R died within 30 days of onset of bacteremia (30-day all-cause mortality, 100%)
AbdulWahab et al. [23], Qatar	To describe the frequency of MDR *P. aeruginosa* recovered from the lower respiratory samples of pediatric and adult cystic fibrosis patients, and its antibiotic resistance pattern to commonly used antimicrobial agents including β-lactams, aminoglycosides, and fluoroquinolones	61 (12 MDR)	A total of 61 *P. aeruginosa* samples were isolated from 30 cystic fibrosis patients from 20 families. Twelve (19.7%) sputum samples (seven non-mucoid and five mucoid) were MDR from five patients with moderate-to-very severe lung disease. The MDR *P. aeruginosa* showed the highest resistance to GEN, AMK and FEP (100%), followed by 91.7% to CIP. None of the isolates were resistant to CST during the study
Sid Ahmed et al. [24], Qatar	To assess the prevalence and antimicrobial susceptibility patterns of MDR *P. aeruginosa* from 5 major hospitals in Qatar	2533 (205 MDR)	The overall prevalence of MDR *P. aeruginosa* was 8.1%; most MDR isolates were from patients exposed to antibiotics during 90 days prior to isolation (85.4%, 177/205), and the infections were mainly hospital-acquired (95.1%, 195/205) with only 4.9% from the community. The majority of MDR isolates were resistant to FEP (96.6%, 198/205), CIP, TZP (91%, 186/205), and MEM (90%, 184/205). Patient comorbidities with MDR *P. aeruginosa* were diabetes mellitus (47.3%, n = 97), malignancy (17.1%, n = 35), end-stage renal disease (13.7%, n = 28) and heart failure (10.7%, n = 22)
Sid Ahmed et al., [25], Qatar	To describe how implementation of an institutional multimodal antimicrobial stewardship program affected the susceptibility of *P. aeruginosa*, the prevalence of MDR *P. aeruginosa* and antibiotic use in the hospital setting	6501 (525 MDR)	The overall prevalence of MDR *P. aeruginosa* was 8.1%, and the yearly prevalence decreased from 9.0% (166/1844) in 2015 (pre-implementation) to 5.5% (122/2234) in 2017 (post-implementation) (*P* = 0.019). Likewise, resistance of MDR *P. aeruginosa* decreased to TZP (90.4–80.3%), MEM (89.2–86.9%), CIP (91–88.5%) and AMK (58.4–47.5%). Studied antimicrobial consumption also decreased by 23.9% post-implementation (*P* = 0.008). The yearly consumption of MEM (*P* = 0.012), TZP (*P* < 0.001) and CIP (*P* = 0.015) significantly decreased from 2014 to 2017
Sid Ahmed et al., [26], Qatar	To investigate the in vitro activity of CZA and C/T against clinical isolates of MDR *Pseudomonas aeruginosa* from Qatar, as well as the mechanisms of resistance	205 MDR (of which 10 were sequenced)	Of all MDR *P. aeruginosa*, susceptibility was 68.8% to CZA, 62.9% to C/T, 59.0% to CZA and C/T, and 27.3% to neither. Less than 50% of XDR isolates were susceptible to CZA or C/T. The 10 sequenced isolates belonged to six different STs and all produced AmpC and OXA enzymes; 5 (50.0%) produced ESBL and 4 (40.0%) produced VIM enzymes. Non-susceptibility to CZA and C/T was largely due to the production of ESBL and VIM enzymes
Sid Ahmed et al., [27], Qatar	To identify the predominant STs and β-lactamase genes in clinical isolates of MDR-*P. aeruginosa* from Qatar	75 MDR	The highest susceptibility was to CZA (48%), followed by C/T (40%). Most isolates possessed Class C and/or Class D β-lactamases (96% each), while MBLs were detected in 26.7%. Eight (40%) MBL-producers were susceptible to ATM and did not produce any concomitant ESBLs. High-risk ST235 (21.3%), ST357 (10.7%), ST389 and ST1284 (8% each) were most frequent. Most ST235 isolates (93.8%) were resistant to all tested β-lactams and had an MDR phenotype
Tawfik et al. [28], Saudi Arabia	To determine the prevalence rate of classes A, B and D β-lactamases among extended-spectrum cephalosporin-non-susceptible *Pseudomonas aeruginosa* clinical isolates from burned patients	156 (35 CAZ-R were tested for EBSLs and MBLs)	The CAZ resistance rate was 22.4%. CAZ-R isolates had resistance rates to piperacillin, TZP, FEP, ATM, IPM, AMK, GEN and CIP of 100%, 71.1%, 88.6%, 48.6%, 70.0%, 82.5%, 87.5%, and 90.0% respectively. No resistance was detected to polymyxine B. The prevalence of ESBL and MBL in CAZ-R *P. aeruginosa* was 69.4% and 42.9%, respectively. VEB-1 (68%) and OXA-10 (56%) are the predominant ESBL genes in CAZ-R *P. aeruginosa* and *bla*_VIM_ is the dominant MBL gene (100%). OXA-10 like gene was concomitant with VEB, GES and/or VIM but PER was not detected
Al-Agamy et al. [29], Saudi Arabia	To profile ESBLs and MBLs in clinical isolates of *P. aeruginosa* resistant to CAZ at a hospital in Riyadh	200 (39 CAZ-R were tested for EBSLs and MBLs)	Resistance was highest to GEN (41%), ticarcillin (35%), piperacillin (32.5%), and CIP (30%). All isolates were sensitive to CST. Among the 39 CAZ-R isolates, 59.0% were ESBL positive, of which 87.0% carried *bla*_VEB_ and 21.7% carried *bla*_GES_ genes. Among the 39 CAZ-R isolates, 41.0% had MBLs, all of which carried *bla*_VIM_ genes. The collection was not dominated by any single clone. This dominance of acquired CAZ-inactivating β-lactamases rather than being attributable to AmpC and efflux
Somily et al. [30], Saudi Arabia	To examine susceptibility of *P. aeruginosa* and *Acinetobacter baumannii* against carbapenems along with CST and tigecycline as alternative therapeutic options	33 MDR	Most MDR *P. aeruginosa* were resistant to IPM (90.9%), and MEM (81.8%), with only 39.4% resistant to doripenem. Colistin had excellent activity against *P. aeruginosa* (93.9% susceptibility). Among the carbapenems, doripenem was found to be the most potent against *P. aeruginosa,* and CST proved to be an effective alternative antimicrobial agent for treatment of *P. aeruginosa*.
Memish et al. [31], Saudi Arabia	To report the first national molecular characterization of carbapenemase production among the main Gram-negative bacteria in the Kingdom, namely members of the Enterobacteriaceae family and the non-fermenters *P. aeruginosa* and *A. baumannii*	39 IPM-R, MEM-R and CAZ-R (11 CP)	VIM is the most prevalent MBL in *P. aeruginosa*, in 8/11 CP isolates, with *bla*_VIM-2_ detected in 54.5%. We detected MBLs in only 20% of the CR *P. aeruginosa*, suggesting that resistance in the majority of the isolates was due to other mechanisms
Khan & Faiz [32], Saudi Arabia	To determine the pattern of antimicrobial resistance of *P aeruginosa*	121	Overall drug resistance was low to moderate to commonly used anti-pseudomonal drugs (4.9–30.6%). Significantly less resistance was exhibited by TZP (4.9%; *P* < 0.05) and MEM showed significantly high resistance (30.6%; *P* < 0.05) as compared to other antibiotics, followed by ticarcillin (22.3%) and IPM (19%), irrespective of the site of infection. Antibiotics with < 10% resistance were FEP (8.3%), AMK (7.4%) and TZP (4.9%). Although, data varied between hospitals, MEM and ticarcillin had the highest drug resistance In all hospitals. Multidrug resistance was 10.7%. This pattern of resistance indicates probable overuse of broad-spectrum antibiotics like carbapenems
Ibrahim [33], Saudi Arabia	To determine the distribution and resistance profiles of Gram-negative bacteria in ICUs at King Abdullah Hospital in Bisha, Saudi Arabia	69	*P. aeruginosa* showed low to moderate resistance rates for aminoglycosides (18.8% to AMK; 20% to tobramycin, 31.7% to GEN). Higher resistance rates were observed to cephalosporins, ATM, MEM, piperacillin, and TZP. Multidrug resistance was 60.9%. *P. aeruginosa* was commonly isolated from ICU infections in this tertiary hospital
Alhussain et al. [34], Saudi Arabia	To determine the risk factors, antimicrobial susceptibility pattern and patient outcomes of *P. aeruginosa* infection in ICU	90	The study included 90 cases and 90 controls. Cases had significantly higher mean (SD) ICU days stay compared with controls; 37.7 (37.8) and 8.37 (8.7) respectively; *P* = 0.001. Resistance was highest to IPM (41.1%) and MEM (27.8%), while 36.7% were MDR. Mortality was similar in both groups: 54 (60.0%) cases and 51 (56.7%) controls; *P* = 0.650. In the final multivariate model, factors independently associated with *P. aeruginosa* were ICU duration, previous surgery, having invasive indwelling devices (tracheostomy tube and urethral catheter), previous surgery and duration of stay in the ICU, and prior use of aminoglycosides
Bukhari et al. [35], Saudi Arabia	To reduce VAP incidence rate, lessen the cost of care, and correlate VAP bundles compliance with VAP incidence rate	Not reported	The predominant pathogen was *P. aeruginosa*, (30.8% of all isolates), of which 93.1% were MDR
Ayoub Moubareck et al. [36], UAE	To investigate mechanisms of carbapenem resistance and genetic relatedness of *P. aeruginosa* isolates recovered in Dubai hospitals	37 (carbapenem-NS)	Of 1969 *P. aeruginosa* isolated during the study period, 471 (23.9%) showed reduced carbapenem susceptibility. Of these, 37 were analyzed and 32% of them produced VIM-type MBLs, including VIM-2, VIM-30, VIM-31, and VIM-42, while GES-5 and GES-9 co-existed with VIM in 5.4% of isolates. Outer membrane impermeability was observed in 73% of isolates and 75.6% displayed overproduced MexAB-OprM. Sequencing revealed one large clone including most CP isolates indicating clonal dissemination. This is the first study on carbapenem NS *P. aeruginosa* from Dubai to show VIM production as well as outer membrane permeability and efflux systems as resistance mechanisms
Alatoom et al. [37], UAE	To compare the activity of C/T and CZA against 120 bacterial strains, including ESBL-producers, CR Enterobacteriaceae, and *P. aeruginosa*, isolated from patients admitted to Cleveland Clinic Abu Dhabi, UAE	31	Twenty-nine (94%) *P. aeruginosa* isolates were susceptible to CZA (MIC_50_, 1.5 µg/ml), whereas 30 (97%) isolates were susceptible to C/T (MIC_50_, 0.75 µg/ml). Overall, C/T and CZA showed comparable activity against *P. aeruginosa*
Sid Ahmed et al. [38], Qatar	To investigate the clinical impact and molecular epidemiology of MDR *P. aeruginosa* in Qatar over a 3-year period	8892 (525 MDR, of which 78 were sequenced)	The overall prevalence of MDR *P. aeruginosa* was 5.9% (525/8892), with the yearly prevalence ranging from 8.1% in 2014 to 4.8% in 2017. MDR isolates demonstrated > 86% resistance to FEP, CIP, MEM and TZP, but 97.5% susceptibility to CST. There were 29 different STs: 20.5% ST235, 10.3% ST357, 7.7% ST389, and 7.7% ST1284. ST233 was associated with bloodstream infections and increased 30-day mortality. All ST389 isolates were obtained from patients with cystic fibrosis
Al-Agamy et al. [39], Saudi Arabia	To describe various molecular and epidemiological characters determining antibiotic resistance patterns in *P. aeruginosa* isolates	34 (CR)	All isolates were CAZ-R. VEB-1 (47.1%) and OXA-10 (41.2%) were the most prevalent ESBL and penicillinase, respectively. VIM-1, VIM-2, VIM-4, VIM-11, VIM-28, and IMP-7 were found in MBL-producers. A decrease in outer membrane porin gene (*oprD*) expression was seen in nine isolates, and an increase in efflux pump gene (*MexAB*) expression was detected in five. Six serotypes (O:1, O:4, O:7, O:10, O:11, and O:15) were found among the 34 isolates. The predominant serotype was O:11 (16 isolates), followed by O:15 (nine isolates). These results revealed diverse mechanisms conferring carbapenem resistance to *P. aeruginosa* isolates from Saudi Arabia
Abdalhamid et al. [40], Saudi Arabia	To detect the prevalence of gastrointestinal tract colonization of CR Enterobacteriaceae and CR *P. aeruginosa* in patients admitted to intensive care units in Saudi Arabia	13 (CR)	An NDM type gene was detected in four CR *P. aeruginosa* isolates and a VIM type gene in one isolate. *AmpC* overexpression was detected in eight CR *P. aeruginosa* isolates. It is possible that *ampC* overexpression was due to overuse of β-lactam agents such as CAZ, which can cause derepression of *ampC*
Somily et al. [41], Saudi Arabia	To estimate the prevalence and resistance trends of isolated non-fermenting Gram-negative bacteria in Saudi Arabia	73,728	*P. aeruginosa* was the most common Gram-negative pathogen isolated. Resistance trends of *P. aeruginosa* were increasing for ATM (absolute increase during the study was 17.3%), IPM (12.3%), and MEM (11.6%). Resistance trends were decreasing for netilmicin (absolute decrease during the study was − 10.0%), AMK (− 5.9%), and tobramycin (− 5.0%). The resistance trends of other tested drugs were generally stable with < 5% change. These included CIP, TZP, CAZ, levofloxacin, FEP, CST, and GEN
Al-Tawfiq et al. [42], Saudi Arabia	To present antibiotic resistance pattern of Gram-negative bacteria over 6 years (2013–2018) in a hospital in Saudi Arabia	4210	Overall susceptibility of *P. aeruginosa* to CAZ was 81–92%, and was 84–91% for FEP, 70–82% for IPM, 92–98% for AMK, and 72–86% for CIP. The susceptibility of *P. aeruginosa* decreased over time (2013–2018) to ceftriaxone, CAZ, and MEM but remained stable to FEP, AMK, CIP, and slightly improved to GEN and trimethoprim-sulfamethoxazole

*AMK* amikacin, *AmpC* ampicillin class C, *ATM* aztreonam, *CAZ* ceftazidime, *CIP* ciprofloxacin, CP carbapenemase-producing, *CR* carbapenem-resistant, *CST* colistin, *C/T* ceftolozane-tazobactam, *CZA* ceftazidime-avibactam, *FEP* cefepime, *GEN* gentamicin, *ICU* intensive care unit, *IMP* imipenemase, *IPM* imipenem, *MBL* metallo‑β‑lactamase, *MDR* multidrug-resistant, *MEM* meropenem, *MIC* minimum inhibitory concentration, *NS* non-susceptible, *OXA* oxacillinase, *PDR* pandrug-resistant, *R* resistant, *SD* standard deviation, *ST* sequence type, *TZP* piperacillin-tazobactam, *UAE* United Arab Emirates, *VEB* Vietnamese extended-spectrum β-lactamase, *VIM* Verona integron-encoded metallo-β-lactamase, *XDR* extensively drug-resistant

**Table 2:** Rates of antimicrobial resistance or non-susceptibility among *P. aeruginosa* from Kuwait, Qatar, Saudi Arabia, and the UAE (2011–2021)

Country/antimicrobial agent	% resistant (Kuwait, Saudi Arabia, the UAE) or non-susceptible (Qatar) *P. aeruginosa* of total^a^										
	2011	2012	2013	2014	2015	2016	2017	2018	2019	2020	2021
Kuwait^b^											
AMK	–	1.9	10.8	8.9	12.2	9.6	10.5	15.8	14.8	10.9	5.1
CAZ	–	7.4	20.0	22.8	18.7	17.6	25.2	19.7	27.4	24.0	22.5
FEP	–	7.4	20.0	17.8	14.6	15.4	21.7	18.4	21.5	20.9	13.0
LVX	–	18.5	21.5	20.8	25.2	26.5	28.0	26.3	32.6	20.2	18.1
MEM	–	24.1	30.8	26.7	23.6	35.3	34.3	35.5	28.9	23.3	16.7
TZP	–	7.4	18.5	22.8	16.3	16.2	20.3	18.4	20.0	18.6	14.5
Total isolates tested (*N*)	1	54	65	101	123	136	143	76	135	129	138
Qatar^c^											
AMK	5	6	7	7	3	0	4	5	2.6	10.3	0.0
CAZ	25	25	26	26	16	26	27	24	26.3	51.3	43.6
FEP	26	24	23	20	22	25	34	30	23.7	46.2	46.2
LVX	–	–	–	–	–	–	–	–	21.1	43.6	48.7
MEM	26	33	34	22	14	38	37	30	36.8	69.2	56.4
TZP	15	26	28	22	22	31	35	28	28.9	56.4	48.7
Total isolates tested (*N*)	390	383	213	155	1668	38	80	99	38	39	39
Saudi Arabia^d^											
AMK	13.2	5.0	35.0	–	0.0	5.0	–	7.7	5.1	4.9	0.0
CAZ	23.7	35.0	50.0	–	10.5	15.0	–	23.1	12.8	17.1	5.9
FEP	13.2	30.0	50.0	–	5.3	10.0	–	15.4	15.4	9.8	2.9
LVX	23.7	50.0	55.0	–	5.3	10.0	–	26.9	23.1	17.1	5.9
MEM	10.5	40.0	50.0	–	21.1	15.0	–	30.8	25.6	19.5	2.9
TZP	10.5	35.0	30.0	–	5.3	10.0	–	11.5	12.8	14.6	2.9
Total isolates tested (*N*)	38	20	20	–	19	20	–	26	39	41	34
UAE^e^											
AMK	4.7	3.8	4.7	4.9	5.0	5.8	4.9	3.9	3.5	2.9	–
CAZ	7.7	6.7	8.5	9.6	10.0	10.2	10.3	8.5	8.3	8.5	–
FEP	7.0	5.3	6.4	7.2	8.0	8.4	7.8	6.5	6.2	6.0	–
LVX	–	–	–	–	–	–	–	–	–	–	–
MEM	10.2	9.1	12.0	14.7	14.4	16.0	14.5	11.0	10.7	10.3	–
TZP	10.5	11.1	10.6	13.5	13.3	13.7	11.9	6.9	6.9	6.7	–
Total isolates (*N*)	1753	2030	2521	3174	4132	4913	6445	7989	8989	9402	–

*AMK* amikacin, *CAZ* ceftazidime, *FEP* cefepime, *LVX* levofloxacin, *MEM* meropenem, *TZP* piperacillin-tazobactam, *UAE* United Arab Emirates

–, no data collected for these years or agents

^a^Rates are presented as the percentage of resistant or non-susceptible *P. aeruginosa* (determined using CLSI breakpoints [64]) of the total isolates collected. Percentages not given when *N* < 10 isolates. All rates are presented to 1 decimal place, with the exception of the non-susceptibility data from Qatar for 2011–2018 [56]

^b^Kuwait resistance data from the ATLAS database [53]

^c^Qatar 2011–2018 non-susceptibility data provided by Hamad Medical Corporation [56]. 2019–2021 non-susceptibility data from the ATLAS database [53]

^d^Saudi Arabia resistance data from the ATLAS database [53]

^e^UAE resistance data provided by UAE MOHAP [57]

### Studies Included in This Review

**Fig. 1:**
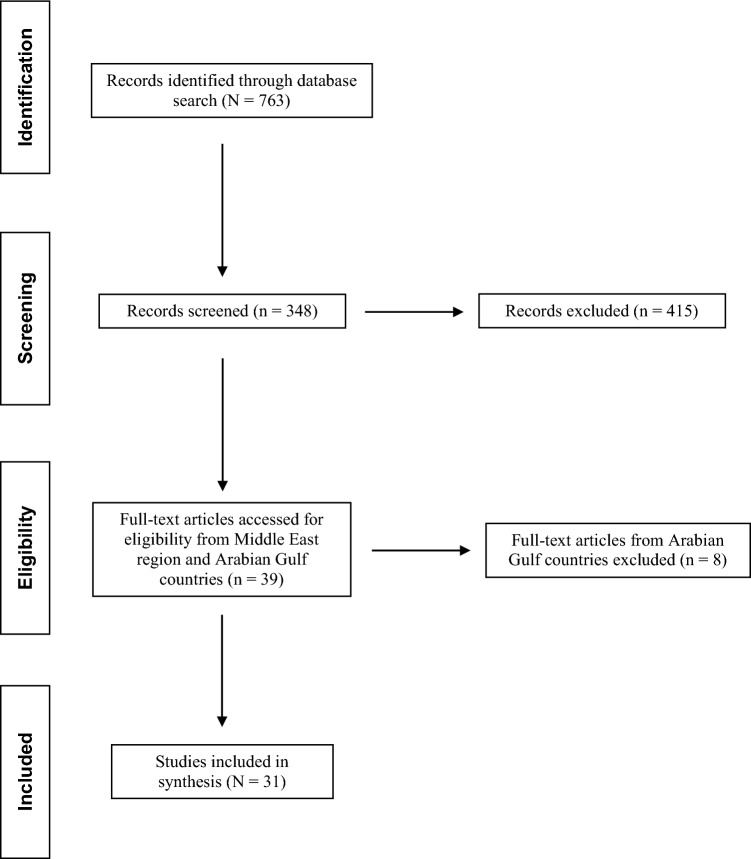
Flow chart for selection of publications on *P. aeruginosa* from the Arabian Gulf countries and other countries/regions (2010–2021)

Following initial identification and screening of publication titles, 34 full-text articles included data on the Middle East region [8, 9, 12–15, 50] or the Arabian Gulf countries (Bahrain, 2; Kuwait, 2; Oman, 3; Qatar, 6; Saudi Arabia, 12; and the UAE, 2) [16–34, 36–42] (Fig. 1). For the Arabian Gulf countries, there were 22 prospective studies (Bahrain: [16, 17]; Kuwait: [18]; Qatar: [23–27, 38]; Saudi Arabia: [28–32, 34, 35, 39–42]; and UAE: [36, 37]) and 5 retrospective studies (Kuwait: [19]; Oman: [20–22]; and Saudi Arabia: [33]). Several studies included data on other species and were presented separately from the *P. aeruginosa* subset (Kuwait: [18, 19]; Oman: [20–22]; and UAE: [37]).

Across the 26 Arabian Gulf studies with documented isolate numbers, a total of 101,839 *P. aeruginosa* isolates contributing data on antimicrobial resistance were distributed as follows: Bahrain, 100 isolates (0.1%) [16, 17]; Kuwait, 150 isolates (0.1%) [18, 19]; Oman, 2620 isolates (2.6%) [20–22]; Qatar, 18,267 isolates (17.9%) [23–27, 38]; Saudi Arabia, 78,702 isolates (77.3%) [28–34, 39–42]; and the UAE, 2000 isolates (2.0%) [36, 37]. These studies were most frequently set in hospital wards (50.0%), followed by a mixture of hospital wards, community settings and ICUs (34.6%). Most studies collected isolates from a variety of culture sources associated with infection (76.9%; including blood, lower respiratory tract, and skin and soft tissue), with only three studies (11.5%) including a proportion of isolates associated with no infection (from colonized patients). Automated antimicrobial susceptibility testing was most frequently used (57.7%), followed by disk diffusion (23.1%), and broth microdilution methodology or E-test (3.8% each). Almost all of these studies used CLSI breakpoints to interpret MIC results (92.3%).

### Antimicrobial Surveillance Data from the ATLAS Program

In addition to the published studies that met the eligibility criteria, this review included global antimicrobial surveillance data from the Antimicrobial Testing Leadership and Surveillance (ATLAS) program (accessible through a publicly available database [53]). The ATLAS program is industry-sponsored and aims to monitor and assess the in vitro activities of selected antimicrobial agents against clinical bacterial isolates collected from hospitalized patients worldwide. The ATLAS program annually collects a prerequisite number of nonduplicate bacterial isolates of clinically significant species (one isolate per species per patient) from documented infection types (intra-abdominal, urinary tract, skin and soft tissue, lower respiratory tract, and bloodstream) [8, 9, 12].

**Fig. 2:**
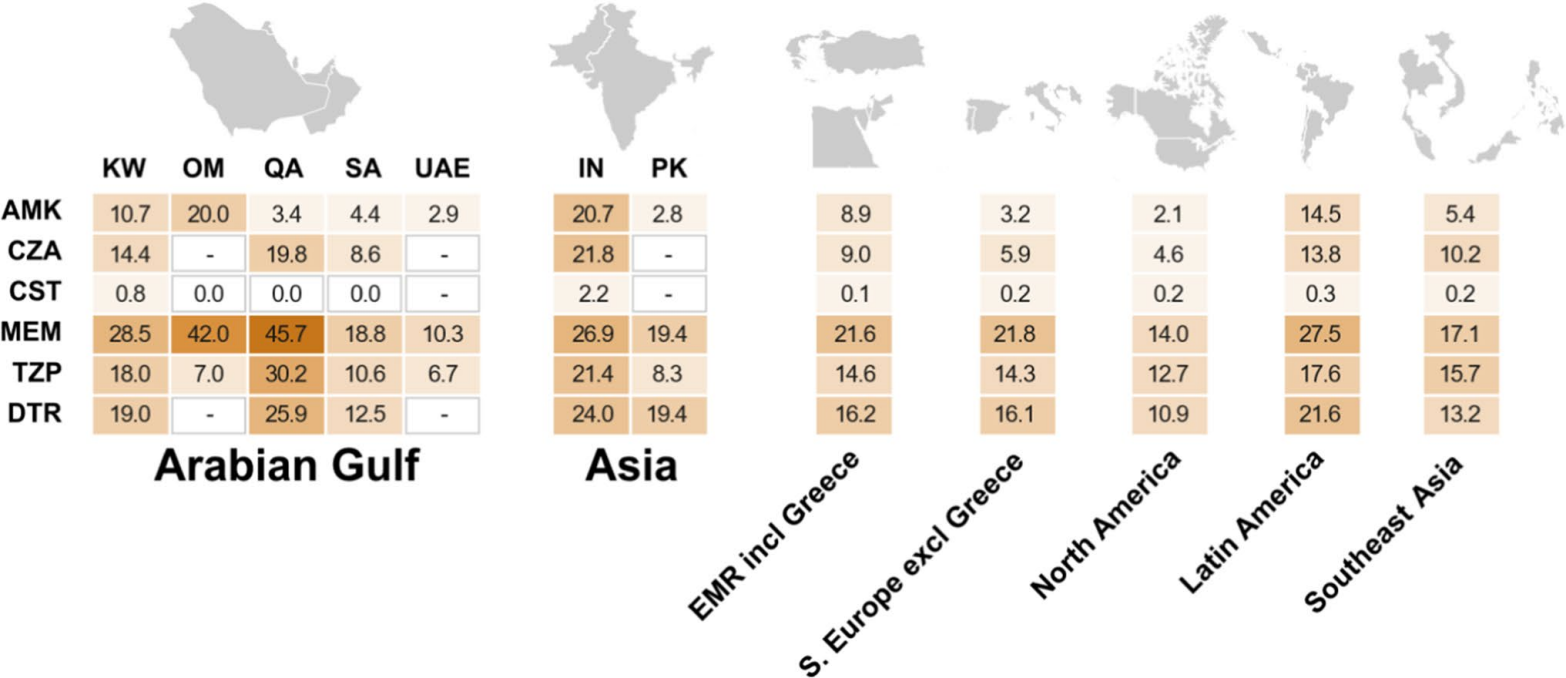
Rates of antimicrobial resistance (resistance data from the ATLAS database [53], except for the UAE [57]) or nonsusceptibility (non-susceptibility data from Oman presented to 1 decimal place but presented to the nearest whole number in the publication [20]) and difficult-to-treat resistance (DTR data from the ATLAS database [53]) among *P. aeruginosa* (2016–2021). Rates of antimicrobial resistance or non-susceptibility were presented as the percentage of resistant or non-susceptible *P. aeruginosa* (determined using CLSI breakpoints [64]) of the total isolates collected. DTR was defined as resistance to >1 antimicrobial in each of the following classes: cephalosporins (ceftazidime, cefepime or ceftriaxone), carbapenems (imipenem, meropenem, doripenem or ertapenem), and quinolones (ciprofloxacin or levofloxacin). Total isolate numbers [years of collection] were: KW, 757 (CZA, CST: 721) [2016–2021]; OM, 2362 [2016 and 2017]; QA, 116 [2019–2021]; SA, 160 (CZA, CST: 140) [2016, 2018–2021]; UAE, 9402 [2020]; IN, 1405 (CZA and CST: 1365) [2016, 2018–2021]; PK, 36 [2016 and 2017]; EMR incl Greece (Greece, Jordan, Israel, and Turkey), 2561 (CZA and CST: 2413) [2016–2021]; S. Europe excl Greece (Croatia, Italy, Portugal, Serbia and Spain), 6185 (CZA and CST: 5063) [2016–2021]; North America (Canada and United States), 5601 (CZA and CST: 4876) [2016–2021]; Latin America (Argentina, Brazil, Chile, Colombia, Costa Rica, Dominican Republic, Mexico, Panama and Venezuela), 6380 (CZA and CST: 5808) [2016–2021]; and Southeast Asia (Malaysia, Philippines, Singapore, Thailand and Vietnam), 2126 (CZA and CST: 2027) [2016–2021]). AMK, amikacin; CZA, ceftazidime-avibactam; CST, colistin; DTR, difficult-to-treat resistance; EMR, Eastern Mediterranean region; excl, excluding; IN, India; incl, including; KW, Kuwait; MEM, meropenem; OM, Oman; PK, Pakistan; QA, Qatar; S., Southern; SA, Saudi Arabia; TZP, piperacillin-tazobactam; and UAE, United Arab Emirates. -, no data collected for these antimicrobial agents in the selected countries

This review presented ATLAS data from other countries and regions, in addition to a total of 1474 isolates from the Arabian Gulf countries (Kuwait, 1101 isolates; Qatar, 116 isolates; and Saudi Arabia, 257 isolates) presented in Table 2 and Fig. 2 [53].

## Antimicrobial Resistance in *Pseudomonas aeruginosa*

### Rates of Antimicrobial Resistance and Phenotypes from the Middle East/Arabian Gulf Region

Figure 2 presents data on the rates of antimicrobial resistance and DTR phenotypes among isolates of *P. aeruginosa* from the Arabian Gulf countries, and other countries or regions [20, 43, 57]. The highest resistance rates observed in each Arabian Gulf country, and the others presented, were to meropenem, ranging from 10.3% (UAE) to >40.0% in Oman and Qatar (higher than the other countries and regions) (Fig. 2). The meropenem resistance rates in Oman and Qatar are reflective of single center data and not necessarily indicative of the resistance status in the country as a whole. Colistin resistance was low (<2.2%) globally (Fig. 2). Amikacin resistance was <5% in Qatar, Saudi Arabia, and the UAE (similar to Southern Europe, North America and Southeast Asia), but higher in Kuwait (10.7%) and Oman (20.0%). There were lower rates of DTR-*P. aeruginosa* (10.9–13.2%) in North America, Saudi Arabia, and Southeast Asia; rates of 16% in Southern Europe and EMR; and rates of 19.0–25.9% elsewhere (data not collected in Oman or the UAE; Fig. 2). Published rates of antimicrobial resistance or susceptibility from the Arabian Gulf countries, and other countries or regions are presented in Supplementary Table 3 [8–10, 12–20, 23, 24, 26–30, 32–34, 36, 37, 41–44].

The rates of antimicrobial susceptibility among a collection of *P. aeruginosa* isolates from Middle East countries (Israel, Jordan, Kuwait, and Saudi Arabia) ranged from 62.8% (levofloxacin) to >90% (amikacin and colistin) [12], according to the European Committee on Antimicrobial Susceptibility Testing (EUCAST) 2020 breakpoints [54]. However, there was no susceptibility breakpoint, and only intermediate or resistance breakpoints, for colistin when using Clinical and Laboratory Standards Institute (CLSI) 2020 breakpoints [55]. Susceptibility to ceftazidime-avibactam and ceftolozane-tazobactam was >90% in the Middle East and Middle East-Africa regions [12, 13].

MDR rates among. *P. aeruginosa* were 30.6% and 38.1%, and the rate with a DTR phenotype was 7.4% for the Middle East region [12, 14]. Among MDR *P. aeruginosa* isolates, cefepime, meropenem and piperacillin-tazobactam susceptibility was 30–50%, which decreased to 0% among DTR isolates [12]. A 2016–2018 ATLAS surveillance study reported that no isolates of carbapenemase-producing *P. aeruginosa* collected in the Middle East-Africa region were resistant to colistin, whereas 32.3% were resistant to aztreonam, and ≥92.3% were resistant to the other tested agents, including amikacin, ceftazidime-avibactam, imipenem and meropenem [9]. These findings reflected the high proportions of MBL-positive isolates that were detected in all regions included in the study [9]. Rates of meropenem non-susceptibility were 25–35% among *P. aeruginosa* collected in the Middle East and Africa [12, 13, 15]. Among meropenem-non-susceptible respiratory *P. aeruginosa* isolates collected from adult intensive-care unit (ICU) patients in the Middle East-Africa region, susceptibility was 68.1% to ceftolozane-tazobactam, 34.9% to ceftazidime, and 28.2% to piperacillin-tazobactam [15].

In the above studies, the range of countries comprising the Middle East-Africa region (including Egypt, Israel, Jordan, Kenya, Kuwait, Lebanon, Morocco, Qatar, South Africa, and Tunisia) were from a wide range of social and economic settings. Antimicrobial susceptibility or resistance data and reports on *P. aeruginosa* isolates from the individual Arabian Gulf countries are described below. The main study findings are summarized in Table 1 and data presented in Supplementary Table 3.

#### Bahrain, Kuwait and Oman

More than 80% of ciprofloxacin-resistant *P. aeruginosa* isolates from Bahrain were also resistant to imipenem, meropenem and piperacillin-tazobactam; however, none were resistant to colistin [16, 17]. Similarly, high resistance rates to meropenem (≥87.5%) and low resistance rates to colistin (2.1%) were observed among MDR *P. aeruginosa* isolates from Kuwait (one of which was pandrug-resistant [PDR]; defined as resistant to all antimicrobial agents [5]) [18]. The rates of susceptibility to amikacin, ceftazidime, ciprofloxacin, imipenem and piperacillin-tazobactam were also notably lower in the study of MDR *P. aeruginosa* from Kuwait, compared with non-MDR isolates [18]. Conversely, *P. aeruginosa* from diabetic foot infections, collected in Kuwait, exhibited susceptibility rates of ≥65.7% to ceftazidime, meropenem, gentamicin, imipenem, and amikacin [19].

In Oman, extended-spectrum β-lactamase (ESBL) enzyme production was only detected in 2 of 2362 *P. aeruginosa* isolates collected [20]. The lowest rate of susceptibility among *P. aeruginosa* from Oman was to meropenem (58.0%), with ≥80.0% of isolates susceptible to amikacin, ceftazidime, ciprofloxacin, gentamicin and piperacillin-tazobactam. All isolates were susceptible to colistin. Another study in Oman found that 8.1% of all MDR isolates from a tertiary care teaching hospital were *P. aeruginosa* [21]. In the same hospital, the rate of CR *P. aeruginosa* in bacteremia was 22% between 2012 and 2016, with a rate of 43% reported in 2015 alone [22].

#### Qatar

Among *P. aeruginosa* from cystic fibrosis patients in Qatar, antimicrobial non-susceptibility ranged from 9.8% (piperacillin-tazobactam) to 41.0% (gentamicin); all isolates were susceptible to colistin [23]. Meropenem non-susceptibility was 11.5% and meropenem resistance was 58.3% among MDR isolates. Among the MDR subset, resistance rates ranged from 50.0% (piperacillin-tazobactam) to 100% (gentamicin, amikacin and cefepime), whereas none of the MDR isolates were resistant to colistin [23].

In a multi-hospital study, the rate of MDR isolates collected in Qatar between 2014 and 2015 was 8.1% (2.4% of which were PDR) [24]. The rate of MDR *P. aeruginosa* at one of these hospitals before the implementation of an antimicrobial stewardship program was 9.0% and was 5.5% after implementation [25]. Most MDR *P. aeruginosa* isolates were resistant to cefepime, ciprofloxacin, piperacillin-tazobactam and meropenem (>90%); in addition, aminoglycoside resistance was 50–75%, and 3.4% were colistin-resistant [25]. Non-susceptibility to ceftazidime-avibactam and ceftolozane-tazobactam was 31.2% and 37.1%, respectively, among MDR *P. aeruginosa* [26]. The authors attributed ceftazidime-avibactam and ceftolozane-tazobactam non-susceptibility rates among the MDR isolates in their study to the production of ESBL and VIM enzymes [26]. Among a smaller subset of eight MDR isolates that produced metallo-β-lactamases (MBLs), but not ESBLs, all were susceptible to aztreonam but resistant to the other agents studied [27].

#### Saudi Arabia

Two studies of *P. aeruginosa* isolates reported low resistance to ceftazidime-clavulanic acid (<10%) and high resistance to gentamicin (41%) [28, 29]. All isolates were susceptible to colistin. In a single-center study of 156 *P. aeruginosa* isolates, 22.4% were ceftazidime-resistant, of which 71.4% were ESBL-producers and 42.9% were MBL-producers [28]. Thirty-nine (19.5%) of 200 clinical *P. aeruginosa* isolates collected at another center were ceftazidime-resistant, most of which were also resistant to other tested agents; however, all isolates were susceptible to colistin [29]. In a single-center study of 33 MDR *P. aeruginosa* isolates, the rates of resistance to colistin and the carbapenems were 6.1% (colistin), 39.4% (doripenem), 81.8% (meropenem), and 90.9% (imipenem) [30]. In a multi-hospital study, the percentage of carbapenemase production among 39 ceftazidime- and carbapenem-resistant isolates of *P. aeruginosa* collected was 28.2% [31].

Another multicenter study statistically compared the overall resistance rates to tested agents among 121 *P. aeruginosa* isolates [32]. The rate of resistance to piperacillin-tazobactam (4.9%) was significantly lower than to ceftazidime, levofloxacin, aztreonam, ciprofloxacin, piperacillin, imipenem, ticarcillin and meropenem (*P* < 0.05). In contrast, the rate of resistance to meropenem (30.6%) was significantly higher than to piperacillin-tazobactam, amikacin, cefepime, gentamicin, ceftazidime, levofloxacin, aztreonam, ciprofloxacin, piperacillin and imipenem (*P* < 0.05). The overall MDR rate was 10.7% [32]. A higher rate of meropenem resistance was observed among respiratory isolates than among other infection types (41.5%; *P* < 0.05).

*P. aeruginosa* isolates from three different ICUs in one hospital showed the lowest rates of resistance to the class of aminoglycosides (amikacin [18.8%], tobramycin [20.0%] and gentamicin [31.7%]), while 30.0% of isolates were resistant to colistin [33]. The study recorded a multidrug resistance rate of 60.9% among *P. aeruginosa* [33]. Similarly, the resistance rates among *P. aeruginosa* isolates from seven ICUs ranged from 8.9% (cefepime) to 41.1% (imipenem). The rate of meropenem resistance was 27.8% [34]. In that ICU study, 36.7% of 90 *P. aeruginosa* isolates collected were MDR [34]. A higher rate of 93.1% MDR *P. aeruginosa* isolates were associated with ventilator-associated pneumonia (VAP) in another ICU study in Saudi Arabia [35].

#### United Arab Emirates

A total of 1969 *P. aeruginosa* were collected in a cross-sectional multicenter study, of which 23.9% of isolates were identified as carbapenem-non-susceptible [36]. Among a subset of 37 carbapenem-non-susceptible isolates that underwent molecular characterization, 10.8% of isolates were MDR and 37.8% were XDR. No study isolates were characterized as PDR because all were susceptible to colistin [36]. A single-center study of 31 *P. aeruginosa* isolates reported rates of resistance ranging from 16.1% (gentamicin) to 51.6% (meropenem) [37]. High susceptibility was reported to ceftazidime-avibactam and ceftolozane-tazobactam among all *P. aeruginosa* isolates (93.5% and 96.8%, respectively).

### Studies on Molecular Resistance Mechanisms from the Middle East/Arabian Gulf Regions

**Fig. 3:**
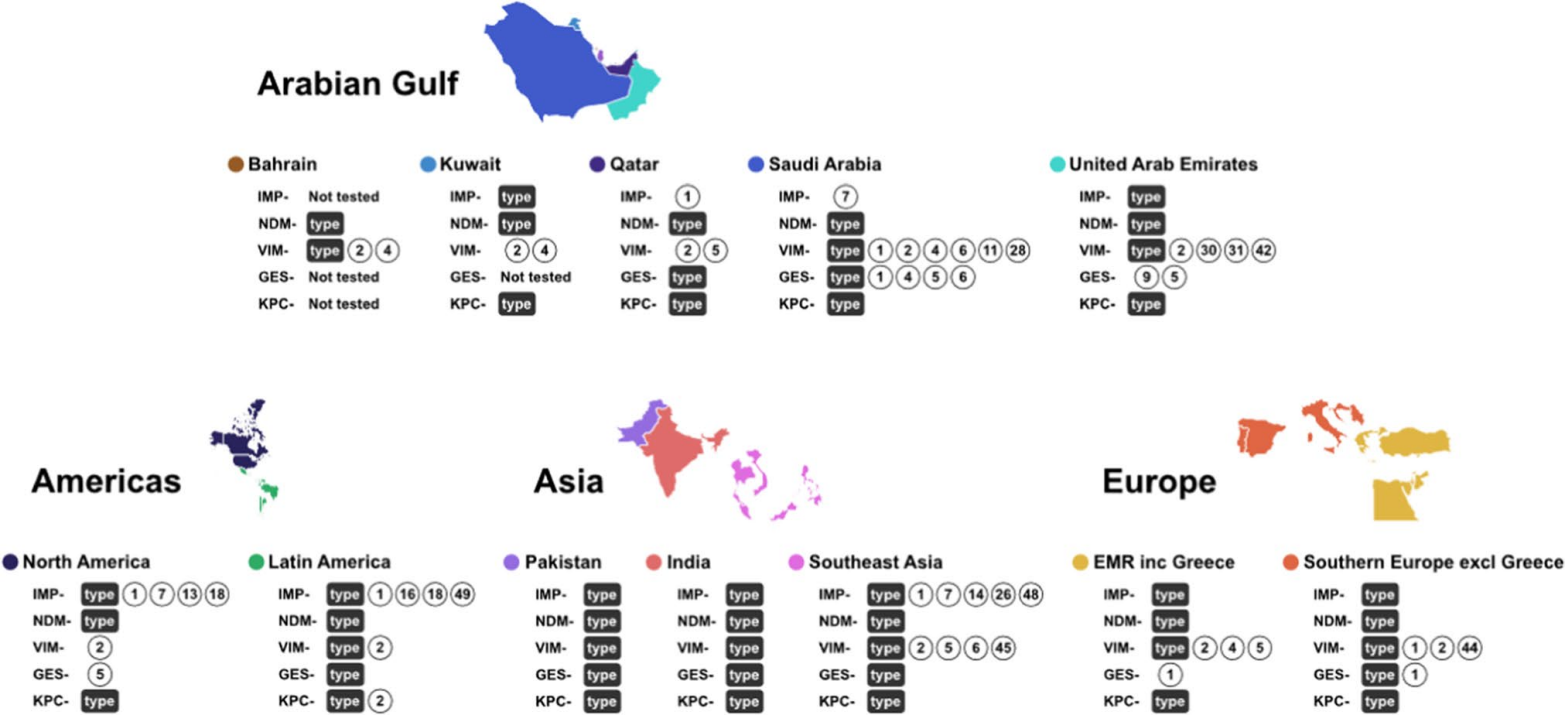
Distribution of β-lactamase genes among *P. aeruginosa* from the Arabian Gulf countries and other countries/regions (2010–2021) (genotype data from each country/region are shown in Supplementary Table 4). Circles show the genotype number of each β-lactamase gene detected in a country or region and ‘type’ represents an identified β-lactamase gene that has not been sub-typed. EMR, Eastern Mediterranean Region; inc, including; excl, excluding; NDM, New Delhi metallo-β-lactamase; VIM, Verona integron-encoded metallo-β-lactamase; IMP, imipenemase; GES, Guiana extended-spectrum; KPC, Klebsiella pneumoniae carbapenemase; and UAE, United Arab Emirates

Figure 3 (data shown in Supplementary Table 4) shows the distribution of β-lactamase genes among *P. aeruginosa* from the Arabian Gulf countries, and other countries or regions. The variety of documented VIM and GES genes appears to be greater in the countries of the Arabian Gulf, compared with the other countries and regions. In contrast, fewer IMP-type genes were reported in the Arabian Gulf, compared with North America, Latin America, and Southeast Asia. Fewer types of NDM and KPC genes were documented among *P. aeruginosa* in the Arabian Gulf and the other countries and regions (Fig. 3, Supplementary Table 4). Molecular characterization studies conducted in the individual Arabian Gulf countries are described below.

#### Bahrain, Kuwait and Qatar

The upregulated expression of genes *mexB*, *mexD*, *mexF* and *mexY* was identified in 8% of tested ciprofloxacin-resistant *P. aeruginosa* isolates [17]. The same isolates also showed a decrease in ciprofloxacin minimum inhibitory concentration (MIC) by the efflux pump inhibitor carbonyl cyanide 3-chlorophenylhydrazone. Among imipenem-resistant *P. aeruginosa* isolates collected from community, hospital, and ICU patients in Bahrain, 52.5% carried MBL genes; mostly VIM-type (90.4%) [16]. One isolate was NDM‑1-positive, and one isolate carried both VIM and NDM‑1 genes.

Data on the β-lactamase genes carried by *P. aeruginosa* isolates in Kuwait are scarce. A global antimicrobial surveillance study that included countries in the Middle East-Africa region showed that 11 isolates of VIM-2-positive and 3 VIM-4-positive *P. aeruginosa* were collected in Kuwait [8].

Among 75 MDR (defined according to [5]) *P. aeruginosa* isolates from two centers in Qatar, 96.0% possessed class C and/or class D β-lactamases, while MBLs were detected in 26.7% of the isolates (*bla*_VIM-2_, *bla*_VIM-5_ and *bla*_IMP-2_) [27]. One (1.3%) isolate co-carried both *bla*_VIM-2_ and *bla*_IMP-1_ and all four β-lactamase classes were present in three (4.0%) isolates. The ESBL gene *bla*_VEB-9_ (formerly known as *bla*_VEB-1a_) was identified as the most frequent ESBL gene (25.3%). High-risk clones ST235 (21.3%), ST357 (10.7%), ST389 and ST1284 (8.0% each) were mostly identified. Most ST235 isolates (93.8%) were resistant to all tested β-lactams. In an additional study from Qatar, sequencing of 78 MDR *P. aeruginosa* revealed 29 different sequence types with the predominance of ST235 (20.5%), followed by ST357 (10.3%), and ST389 and ST1284 (7.7% each) [38]. ST233 was associated with bloodstream infections and increased 30-day mortality, while all ST389 were isolated from cystic fibrosis patients.

#### Saudi Arabia

Two studies found that *bla*_VEB_ genes were predominantly harbored by phenotypically ESBL-positive *P. aeruginosa* isolates (68.0% and 87.0%, respectively) [28, 29]. Tawfik et al. [28] also identified *bla*_OXA-10_ genes in 56.0% of ESBL-producing isolates, followed by *bla*_GES_ in 20.0%. Both studies found that all MBL-producing *P. aeruginosa* isolates carried *bla*_VIM_ genes but neither study found *bla*_PER_, *bla*_TEM_, *bla*_SHV_ or *bla*_CTX-M_ among the ESBL genes, nor *bla*_IMP_, *bla*_GIM_, *bla*_SIM_, *bla*_SPM_ or *bla*_NDM_ among the MBL genes [28, 29]. Tawfik et al. [28] suggested that the higher percentage of *bla*_VEB_ than *bla*_OXA-10_ and *bla*_GES_ ESBL genes could be due to residents from regions with a higher prevalence of *bla*_VEB_ genes (such as *bla*_VEB-1_ in Southeast Asia) living in Saudi Arabia. In addition, the collection of clinical *P. aeruginosa* isolates was not dominated by any single clone and the identification of multiple *bla* genes did not solely reflect the spread of a single outbreak strain [28, 29].

A subsequent study by Al-Agamy et al. [39] also found that the predominant ESBL was VEB-1 (47.1%), followed by OXA-10 (41.2%) and GES (8.8% [GES-1, GES-4 and GES-6]) among CR *P. aeruginosa* isolates. As previously found, the most common MBLs were VIM-type, but in this later study, IMP-7 was also found [28, 29, 39]. Further analysis showed the downregulation of *OprD* porin gene expression in nine (26.5%) isolates and an upregulation of MexAB efflux pump gene expression in five (14.7%) isolates [39]. Six different serotypes and 14 different pulsotypes were detected; of which all 9 serotype O:15 strains were found to have the same pulsotype (F) and the same mechanism of resistance (OXA-10 and VEB-1a).

A multicenter study investigating the carriage of MBL genes in 39 CR *P. aeruginosa* isolates found that 28.2% were phenotypic carbapenemase-producers, with VIM being the dominant MBL detected (72.7% [VIM-2, VIM-6 and VIM-28]). Three isolates carried the *bla*_GES-5_ gene. No IMP-producing genes were detected among any *P. aeruginosa* isolates [31].

In a study of digestive tract colonization by *P. aeruginosa* in ICU patients, 5 of 13 CR *P. aeruginosa* isolates from rectal swabs were shown to carry MBL carbapenemases (4 with NDM and 1 with VIM) [40]. AmpC overexpression, which has been linked with carbapenem resistance when coupled with porin mutations, was detected in eight isolates of CR *P. aeruginosa*. The overexpression of *bla*_AmpC_ in these isolates may have arisen due to the overuse of β-lactams, such as ceftazidime, which can cause derepression of AmpC. The authors reported that this was the first study in the Arabian Gulf region to document AmpC overexpression in CR *P. aeruginosa* isolates [40].

#### United Arab Emirates

In a cross-sectional survey on carbapenem-non-susceptible *P. aeruginosa*, the VIM-type MBL carbapenemases were most common among a subset of 37 that were analyzed (32.4% [VIM-2, VIM-30, VIM-31 and VIM-42]) [36]. A single VIM-2-carrying isolate was also GES-9-positive, while one VIM-42-positive isolate co-carried GES-5. No other investigated MBL genes were detected, nor were KPC carbapenemases. Outer membrane impermeability was observed in 73% of isolates and 75.6% displayed overproduced MexAB-OprM efflux pump. Seven distinct clones were identified, one of which comprised 81.1% of isolates across all hospitals (including 11 out of the 12 isolates that were VIM- and GES-positive), suggesting clonal dissemination [36].

### Changes in Antimicrobial Resistance Since 2011 in the Arabian Gulf Region

#### Kuwait

Between 2012 and 2021, there were net increases of antimicrobial resistance to amikacin (3.2%), ceftazidime (15.1%), cefepime (5.6%), and piperacillin-tazobactam (7.1%) [53] (Table 2). Resistance to levofloxacin remained stable (−0.4% net change), and to meropenem (−7.4% net change). Following 2019, the rates to all agents appeared to decrease annually.

#### Qatar

Between 2011 and 2018, the rates of non-susceptibility to amikacin and ceftazidime remained stable (net changes, 0.0% and −1.0%, respectively), whereas non-susceptibility to cefepime, meropenem and piperacillin-tazobactam increased (by 4.0%, 4.0%, and 13.0%, respectively) [56] (Table 2). ATLAS 2021 data showed higher rates of non-susceptibility than in 2011 for all tested agents except amikacin [53] (Table 2).

#### Saudi Arabia

In a 6-year, multicenter national antimicrobial surveillance study, the trend was increased resistance to aztreonam, imipenem and meropenem, with overall percentage increases during the study period of 17.3%, 12.3% and 11.6%, respectively [41]. Overall percentage decreases in resistance during the study period were shown to netilmicin, amikacin and tobramycin (by 10.0%, 5.9% and 5.0%, respectively). Resistance to the other antimicrobials on the test panel (including ciprofloxacin, piperacillin-tazobactam, ceftazidime, levofloxacin, cefepime, colistin and gentamicin), remained stable with <5% overall change. In another 6-year antimicrobial surveillance study, the susceptibility of. *P. aeruginosa* to ceftriaxone, ceftazidime and meropenem decreased over the study period, but remained stable to cefepime, amikacin, ciprofloxacin, and increased to gentamicin and trimethoprim-sulfamethoxazole [41].

ATLAS antimicrobial resistance data were not collected in 2014 or 2017, but resistance rates to all tested agents were higher in 2011–2013 than 2018–2021. Between 2018 and 2021, there appeared to be a decrease in resistance to all agents tested [53] (Table 2).

#### United Arab Emirates

In the UAE, *P. aeruginosa* showed a horizontal trend for resistance to fluoroquinolones, and to third- and fourth-generation cephalosporins, and showed a decreasing trend for resistance to aminoglycosides. Amikacin and piperacillin-tazobactam resistance decreased by 1.8% and 3.8%, respectively, and resistance to ceftazidime, cefepime and meropenem remained stable (net changes, 0.8%, −1.0%, and 0.1%, respectively) (Table 2). Nevertheless, from 2016 to 2020, both carbapenems, imipenem and meropenem, showed a decreasing trend of resistance. The percentage of MDR, XDR, and possible PDR isolates generally declined from 2010 to 2020 [57].

## Discussion

The studies above describe important published findings on antimicrobial resistance among isolates of *P. aeruginosa* in the Arabian Gulf countries since 2010. More information is now known about the genes and mechanisms driving resistance among *P. aeruginosa* in these countries, and about the antimicrobial agents that could be used against *P. aeruginosa* infections in a hospital or ICU setting. Recent data (2016–2021) on antimicrobial resistance among clinical isolates of *P. aeruginosa* show that resistance to meropenem was highest (10.3–45.7%) among the agents presented [53]. In Kuwait, there was an overall increase in resistance rates from 2012 to 2021 to most antimicrobial agents presented, although levofloxacin and meropenem resistance appeared to fluctuate [53]. Data collected over the last 12 years from Saudi Arabia showed an increase in resistance to aztreonam, imipenem and meropenem, and a decrease in susceptibility of. *P. aeruginosa* to ceftriaxone and ceftazidime [41, 42]. The reported increase in resistance rates is alarming, and continued surveillance is needed to monitor these upward trends. The significantly higher rate of resistance to meropenem than to other tested agents among *P. aeruginosa* in one study from Saudi Arabia was suggested to be due to over-prescribing of broad-spectrum antimicrobial agents, such as carbapenems [32]. Prior antibiotic treatment was found to be associated with MDR *P. aeruginosa* infections, according to studies in Saudi Arabia and Qatar [24, 34]. Further studies are warranted to correlate antimicrobial use in *P. aeruginosa* infections with the current resistance profiles in the Arabian Gulf countries.

In treatment guidance from the IDSA, DTR among *P. aeruginosa* is defined as non-susceptibility to piperacillin-tazobactam, ceftazidime, cefepime, aztreonam, meropenem, imipenem-cilastatin, ciprofloxacin and levofloxacin [8]. The most common MDR profile among *P. aeruginosa* in the study by Karlowsky et al. [14] was non-susceptibility to aztreonam, ceftazidime, cefepime, ciprofloxacin, imipenem and piperacillin-tazobactam. These resistance profiles leave few antimicrobial agents with known activity against *P. aeruginosa*, one of which is colistin. Colistin demonstrated the highest antimicrobial activity among the studies reported in this review, including those with subsets of antimicrobial-resistant phenotypes; however, owing to the increased use of colistin as a last-resort agent for infections caused by MDR and XDR strains, colistin resistance among MDR and XDR *P. aeruginosa* is emerging worldwide. Thus, resistance mechanisms in these antimicrobial-resistant phenotypes are being investigated [58–60]. Therefore, colistin resistance among *P. aeruginosa* requires continued surveillance and suitable monitoring systems to report the dissemination rate of these resistance genes.

It has been suggested that the Middle East region could act as a secondary reservoir for NDM carbapenemases, on account of population flow to the Middle East from countries of the Asian subcontinent, such as India or Pakistan [61, 62]. While NDM- and VIM-type β-lactamase genes are predominant in the Asian subcontinent, only four isolates included in this review were found to harbor NDM-type genes (one from Bahrain and three from Saudi Arabia) [16, 40]. The lack of molecular data and proper representation could be a contributing factor to this observation. Further research and enhanced surveillance efforts are needed to fully understand the distribution and prevalence of NDM-type genes. Although the VIM β-lactamase appears to be dominant in the Arabian Gulf, multiple resistance mechanisms were found to cause carbapenem resistance in most *P. aeruginosa* isolates from Saudi Arabia and the UAE [28, 29, 31, 36, 39]. Multiple clones of MDR *P. aeruginosa* were also identified in Bahrain, Qatar, Saudi Arabia, and the UAE, including ST235, ST233 and ST357 which are deemed as high-risk clones [27, 38]. Their associated MDR phenotypes are a cause for concern. Clonal dissemination was also discussed by Ayoub Moubareck et al. [36] regarding their study findings of seven distinct clones among carbapenem-non-susceptible *P. aeruginosa* isolates from the UAE. This is also concerning as clonal dissemination contributes to the spread of *P. aeruginosa* pathogens that carry β-lactamase genes and are antimicrobial-resistant.

When comparing antimicrobial susceptibility or resistance data from different studies of antimicrobial surveillance or prevalence (whether national, regional or global), it is vital to be aware of the limitations of comparisons made. We express similar limitations to the observations noted by the European Centre for Disease Prevention and Control on inter-country comparisons and national trends of surveillance data on antimicrobial resistance, such as population coverage, sampling, laboratory routines [63]. Some of the most important potential sources of bias are the various protocols for antimicrobial susceptibility testing, the use of guidelines for clinical breakpoints, various isolate sources and numbers of isolates, the sizes of the hospitals (and whether single- or multi-center), and single versus multiple study years. Details on isolate collection and susceptibility testing, where available, for each study are noted in Supplementary Table 2. Data from individual non-national studies may not reflect the trends of the whole country and might generate inaccurate reporting bias for prevalence rates, microbiological characteristics, or mechanisms of genetic resistance. Furthermore, it is important to acknowledge that excluding papers published in Arabic language may limit the scope of the study and the perspectives represented.

Despite these limitations, the sharing of data on the local, national, and international levels of antimicrobial-resistant *P. aeruginosa* may serve to improve public health, inform health policies, provide evidence for developing treatment guidelines, and monitor the trends and spread of resistance. Local epidemiology data can inform the implementation of infection prevention and control, and antimicrobial stewardship programs in the respective healthcare institutions. Together, these factors need to be addressed as a matter of urgency to establish a more comprehensive and representative antimicrobial resistance surveillance system to monitor the threat of the opportunistic pathogen *P. aeruginosa*.

## Supplementary Information

Below is the link to the electronic supplementary material.

Supplementary file1 (DOCX 106 KB)

## Data Availability

The datasets generated and/or analyzed during the current study are included in this published article [and its supplementary information files], or available from the corresponding author on reasonable request.

## Abbreviations

AmpC: Ampicillin class C
bla: β-Lactamase gene
CDC: Centers for Disease Control and Prevention
CLSI: Clinical and Laboratory Standards Institute
CR: Carbapenem-resistant
DTR: Difficult-to-treat resistance
ESBL: Extended-spectrum β-lactamase
EUCAST: European Committee on Antimicrobial Susceptibility Testing
GES: Guiana extended-spectrum
IDSA: Infectious Diseases Society of America
I: Intermediate
ICMR: Indian Council of Medical Research
ICU: Intensive care unit
IMP: Imipenemase
KPC: Klebsiella pneumoniae carbapenemase
MBL: Metallo-β-lactamase
MDR: Multidrug-resistant
mex: Multidrug efflux pump
MIC: Minimum inhibitory concentration
NDM: New Delhi metallo-β-lactamase
NS: Non-susceptible
Opr: Outer membrane porin
OXA: Oxacillinase
PDR: Pandrug-resistant
PER: Plasmid-mediated extended spectrum
R: Resistant
S: Susceptible
SPM: São Paulo metallo-β-lactamase
ST: Sequence type
TZP: Piperacillin-tazobactam
UAE: United Arab Emirates
VAP: Ventilator-associated pneumonia
VEB: Vietnamese extended-spectrum β-lactamase
VIM: Verona integron-encoded metallo-β-lactamase
XDR: Extensively drug-resistant
