# Supplementary material for: Antimicrobial Resistance Profiles of Pseudomonas aeruginosa in the Arabian Gulf Region Over a 12-Year Period (2010–2021)
Source: J Epidemiol Glob Health. 2024 Jun 10;14(3):529–48. doi: 10.1007/s44197-024-00191-y (PMC11442796; doi:10.1007/s44197-024-00191-y)
Supplement: Supplementary file 1 — Supplementary file1 (DOCX 106 KB) [file 44197_2024_191_MOESM1_ESM.docx]

# Supplementary Table 1. Major and mid-search terms and related synonyms

| Topic | Synonyms/keywords |
| --- | --- |
| **1. *Pseudomonas aeruginosa*** | *Pseudomonas*, *P. aeruginosa*, Pseudomonal, multidrug resistance, multidrug-resistant, multi-drug resistant, MDR, drug-resistant |
| **2. Gulf region** | Gulf, Bahrain, Kuwait, Oman, Qatar, United Arab Emirates, UAE, Arab, Emirates, Middle East, Arabic, Arabian, Gulf country/countries, Gulf state(s), global |
| **3. Infections** | Prevalence, incidence, infection rate, type(s) of infections, patient demographics (hospitals vs community), cystic fibrosis, disease burden, hospital stay, mortality, clinical outcome |
| **4. Resistance** | (Antimicrobial) resistance, surveillance, resistance rate(s), resistance mechanism(s), multidrug resistance, multi-drug resistance, multidrug-resistant, multi-drug resistant, MDR |
|  | |

# Supplementary Table 2. Study methodology details for publications on the rates of antimicrobial resistance in *P. aeruginosa* from the Arabian Gulf countries and incorporating the Middle East region (2010–2021)

| **Study reference** | **Source of *P. aeruginosa* isolates** | **Dates of collection** | **Region/Country/Center** | **Method of susceptibility testing and clinical breakpoints used** |
| --- | --- | --- | --- | --- |
| **Kazmierczak et al., 2015 [8]** | Non-duplicate isolates from bloodstream, intra-abdominal, lower respiratory tract, skin and soft- tissue and urinary tract infections. A predefined number of selected bacterial species. | 2012–2014 | Global (Asia-Pacific, Europe, Latin America, the Middle East-Africa [Kenya, Kuwait, Nigeria, South Africa], and North America) | MICs were determined using frozen broth microdilution panels. Panel manufacture, inoculation, incubation, and interpretation were performed according to CLSI guidelines (CLSI documents M07-A9 and M100-S25). |
| **Kiratisin et al., 2021 [9]** | The probable causative pathogen of the documented infection (lower respiratory tract, skin and soft-tissue, urinary tract, bloodstream and intra-abdominal infections). A predefined number collected for each species. | 2016–2018 | Africa/Middle East (Israel, Jordan, Kuwait, Morocco, Nigeria, Saudi Arabia, and South Africa) | Antimicrobial susceptibility testing was performed centrally using frozen reference broth microdilution panels, according to EUCAST guidelines (ISO 20776-1 [2006]). MICs were interpreted using EUCAST (Version 10.0, 2020) breakpoints. |
| **Karlowsky et al., 2021 [12]** | Bloodstream, intra-abdominal, respiratory tract, skin and soft tissue and urinary tract infection specimen sources | 2015–2018 | Middle East (Israel, Jordan, Kuwait, and Saudi Arabia) | Antimicrobial susceptibility testing was performed using CLSI broth microdilution methodology (CLSI M07-A11 and M100-S13). MICs were interpreted using CLSI (M100-S30) and EUCAST (Version 10.0, 2020) MIC breakpoints. |
| **Nichols et al., 2016 [13]** | Clinical isolates (sources not listed) | 2012–2014 | Global (Asia/South Pacific, Europe, Latin America, and Middle East/Africa) | Antimicrobial susceptibility testing was performed following the CLSI standard method for broth microdilution (CLSI M100-S25 and M07-A10) using inhouse-prepared, 96-well panels. MICs were interpreted using CLSI (M100-S25) breakpoints. For CZA (CLSI breakpoints not available for *P. aeruginosa*), MICs were interpreted using FDA breakpoints (S, ≤8 µg/ml; R, ≥16 µg/ml). |
| **Karlowsky et al., 2018 [14]** | Annually-collected consecutive aerobic and/or facultative Gram-negative pathogens cultured from lower respiratory tract (n = 100), intra-abdominal (n = 100) or urinary tract (n = 50), and unknown specimens of unique patients. | 2015–2016 | Global (Africa, Asia, Europe, Latin America, Middle East (Georgia, Israel, Jordan, Lebanon, Saudi Arabia, and United Arab Emirates), United States/Canada, and South Pacific) | Antimicrobial susceptibility testing was performed according to the CLSI reference broth microdilution standard method (CLSI M100-S27 and M07-A10) using custom-made dehydrated 96-well panels. MICs were interpreted by applying current CLSI (M100-S27) breakpoints. For imipenem/relebactam (CLSI breakpoints not available for *P. aeruginosa*), MICs were interpreted using imipenem CLSI breakpoints (S, ≤2 µg/ml; I, 4 µg/ml; R, 8 µg/ml). MDR was defined as NS (i.e., I or R) to ≥3 of the following: AMK, AZT, FEP, CAZ, CIP, CST, IPM and TZP. |
| **Moise et al., 2021 [15]** | Up to 100 consecutive clinically relevant Gram-negative bacilli (one isolate per patient episode) from lower respiratory tract specimens, up to 50 isolates from urinary tract specimens, up to 50 isolates from intra-abdominal specimens, and up to 50 isolates from bloodstream specimens. Respiratory isolates from adult patients with respiratory infections in the ICU were included. | 2018 | Global (Asia Pacific, Europe, Latin America, Middle East/Africa [Israel, Jordan, Kenya, Kuwait, Lebanon, Morocco, Qatar, South Africa, and Tunisia] and North America) | Susceptibility testing was performed by broth microdilution methods using CLSI recommendations (not referenced). Categorical interpretation of susceptibility was defined according to the 2020 CLSI M100 (CLSI M100-S30). |
| **Joji et al., 2019 [16]** | Non-duplicate strains from clinical samples (included community, wards, and ICU patients). Repeat isolates from the same patients were excluded from the study. Isolates were from endotracheal aspirate, swab, blood, respiratory secretions, urine and tissue. | Not reported | Salmaniya Medical Complex, King Hamad University Hospital, and Bahrain Defense Force Hospital, Bahrain | Antimicrobial susceptibility testing was performed by the disc diffusion method on Mueller‑Hinton agar plates and interpreted according to CLSI recommendations (CLSI, 2016) |
| **Al Rashed et al., 2020 [17]** | Non-duplicate isolates collected from patients from swabs, deep tracheal aspiration, endotracheal tube, drain fluid aspiration, urine, sputum, blood and tissues | Not reported | Salmaniya Medical Complex, King Hamad University Hospital, and Bahrain Defense Force Hospital, Bahrain | The susceptibility pattern of the isolates was determined according to the CLSI guidelines (M100-S27) by disc diffusion test |
| **Alfouzan et al., 2018 [18]** | Non-duplicate (single isolate per patient) isolates from blood, respiratory, or urine samples | Not reported | Farwania Hospital, Kuwait | Antimicrobial susceptibility testing was performed by using broth microdilution panels, according to CLSI standards (M07, 2015 and M100, 2018). MDR isolates showed resistance to ≥3 classes of antibiotics by disk diffusion or commercial methods. CST MICs were interpreted as an ECV and expressed as Wild Type (S, ≤2 µg/mL) or Not Wild Type (R, ≥4 µg/mL). |
| **Alhubail et al., 2020 [19]** | Outpatients with diabetic foot ulcers (with and without clinical infection included) from swabs and tissue samples | January–December 2017 | Dasman Diabetes Institute clinics, Kuwait | Identification was done according to CLSI-M50 and CLSI-M100. Antimicrobial sensitivity testing of all isolates was performed on diagnostic sensitivity test plates, according to the Kirby-Bauer method [PMID: 5325707] following the definition of the CLSI (CLSI, 2014). |
| **Al Rahmany et al., 2019 [20]** | A convenience sample of all isolates from outpatient and inpatient skin and soft tissues, urine, respiratory system, blood, patient related devices, stool, CSF, or other body fluids. Duplicate isolates were excluded and samples were excluded per protocol if they fulfiled all the following criteria: linked to same patient identification number, collected within <1 week, and identified the same pathogen with identical, antibiotic susceptibility pattern. | January 2016–December 2017 | Suhar Hospital, Oman | All procedures were performed according to CLSI guidelines (Standards Development Policies and Process 0ctober, 2013). For antibiotic susceptibility of the isolated bacteria, Kirby-Bauer disk diffusion technique was done on Mueller-Hinton agar. All Gram-negative bacteria that were NS to ≥1 antibiotic in ≥3 antimicrobial categories were reported as MDR. |
| **Balkhair et al., 2014 [21]** | Bloodstream infection, pneumonia, urinary tract infection, surgical infection, and others. Not all isolates may represent active infections from the patients. Samples could have been community or nosocomial onset of infection. | January–December 2012 | Sultan Qaboos University Hospital, Oman | Organisms were identified and tested by automated identification and susceptibility system. Susceptibility testing was confirmed by disk diffusion method. MDR was defined according to **[5]**. |
| **Balkhair et al., 2019 [22]** | Blood culture isolates from community-acquired and healthcare-associated bacteremia. The classification of bacteraemia as healthcare-associated or community-acquired was based on hospital infection prevention and control categorization using WHO definitions. Blood cultures that were simultaneously positive for ≥2 of the study isolates were excluded.The source of bacteraemia was not studied. | January 2007–December 2016 | Sultan Qaboos University Hospital, Oman | The presence of carbapenem resistance was detected by commercial automated system. The interpretation of carbapenem susceptibility was based on the CLSI definitions (not reported) as R, S or not tested. *P. aeruginosa* isolates were considered R to carbapenems if R to either IPM or MEM but not ETP. The prevalence of carbapenem resistance was studied in selected isolates from blood cultures. |
| **AbdulWahab et al., 2017 [23]** | Lower respiratory specimens such as sputa, deep pharyngeal swab, or bronchoalveolar lavages were collected from CF inpatients and outpatients | October 2014–September 2015 | Hamad Medical Corporation, Qatar | Identification and antimicrobial susceptibility were performed using an automated system and *E*-test (previously known as Epsilometer test) methods in compliance with CLSI guidelines (M100-S25). MDR was R to all agents in ≥2 classes among: aminoglycosides, β-lactams, and/or fluoroquinolones, CIP). |
| **Sid Ahmed et al., 2019a [24]** | Routine inpatient and outpatient isolates from respiratory tract (7% CF), skin and soft tissue, urine, blood, and other specimens. Isolates from a patient wof the same species and susceptibility pattern isolated within 30 days from sites except blood and sterile body fluid were excluded. Isolates with different susceptibility patterns were considered as new even if isolated within 30 days. Most patients (59%) were colonized (no clinical signs and symptoms of ongoing infection) and 41% were infected. | October 2014–September 2015 | Hamad General Hospital, Rumailah Hospital, National Centre for Cancer Care and Research, and the Heart Hospital, Qatar | Isolates were analysed using an automated system in compliance with the CLSI. When analyses terminated due to a lack of emulsification of mucoid strains, antimicrobial susceptibility was tested manually using MIC test strips. Results were interpreted using the CLSI reference breakpoints (M100-S25). Isolates classified as I or R were defined as NS. MDR was defined as R to ≥1 agent of ≥3 antibiotic classes. |
| **Sid Ahmed et al., 2020a [25]** | Consecutive non-duplicate *P. aeruginosa* isolates from respiratory, skin and soft tissue, urine, blood, and other infection sites in hospitalized patients. A bacterial isolate of the same species and same antimicrobial susceptibility pattern in a patient isolated within 30 days, regardless of the site of isolation, was excluded. Isolates with major differences in susceptibility patterns were considered as new even if isolated within 30 days. | October 2014–September 2017 | Hamad General Hospital, Qatar | The bacterial identification and initial antimicrobial susceptibility testing was done using an automated system, while MIC test strips and the standard reference strains were used for quality control, as per recommendations of the CLSI. Results were interpreted using CLSI reference breakpoints (not reported). MDR was defined according to **[5]**. |
| **Sid Ahmed et al., 2019b [26]** | Clinical isolates from respiratory cultures, skin and soft tissue, urine, blood, sterile body fluids, and vascular line tips | October 2014–September 2015 | Hamad General Hospital, Rumailah Hospital, National Centre for Cancer Care and Research, and the Heart Hospital, Qatar | An automated system was used for bacterial identification and initial antimicrobial susceptibility testing, while MIC test strips were used for controls. Susceptibility reporting was based on current recommendations of the CLSI (M100-S29). For consistency, I and R breakpoint categories were grouped together as NS. MDR was defined according to **[5]**, and also R to CZA and/or C/T. |
| **Sid Ahmed et al., 2020b [27]** | Isolates from skin and soft tissue, urine, respiratory tract, blood and other sample types | October 2014–September 2017 | Hamad General Hospital, Rumailah Hospital and the National Center for Cancer Care and Research, Qatar | Microbiological identification and susceptibility tests were performed by an automated system and manual MIC test strips. Susceptibility reporting was based on CLSI breakpoint recommendations (M100-S30). MDR was defined according to **[5]**. |
| **Tawfik et al., 2012 [28]** | Non-duplicate, non-consecutive isolates from burned patients | 2010 | Prince Salman Hospital, Saudi Arabia | Antimicrobial susceptibility tests were performed using the disc diffusion method recommended by the CLSI guidelines (M7-A7). MICs were by E-test method, according to CLSI recommendations (M7-A7). |
| **Al-Agamy et al., 2012 [29]** | Non-duplicate, non-consecutive clinical isolates from burn, sputum, pus, urine, eye discharge, and blood isolation sites | August–November 2010 | Armed Forces Hospital, Saudi Arabia | Susceptibility testing was by the disc diffusion method of the CLSI (M7-A7). MICs were determined with E-test strips. |
| **Somily et al., 2012 [30]** | Non-duplicate clinical isolates from isolated from a variety of body sites including wounds, respiratory, urine, swabs, tissues, blood, and other sterile body sites. Samples were collected consecutively and one sample from each patient were included. | June–December 2010 | King Khalid University Hospital, Saudi Arabia | MICs were determined by E-test and interpreted as the value at which the inhibition zone intercepted the scale on the E-test strip. Susceptibility test results were interpreted according to the CLSI breakpoint recommendations (M100-S21). For tigecycline (CLSI breakpoints not available for *P. aeruginosa*), results were interpreted as S, ≤2; I, 4, and R, ≥8 mg/L. MDR was CR or R to 3 classes of antimicrobials (PMID: 18068814). |
| **Memish et al., 2015 [31]** | Non-duplicate clinical isolates from skin/wound, blood, urine, stool, respiratory and other specimens. A single Gram-negative species was isolated from a single patient’s specimens, but in some cases, >1 species was isolated from the same patient’s specimens. | January–June 2012 | Asir Central Hospital (Abha); Maternity and Children Hospital (Al-Ahsa); King Fahad Hospital (Al-Ahsa); Dammam Medical Complex (Dammam); Qatif Central Hospital (Qatif); King Fahad Specialist Hospital (Buraidah); Madinah Maternal and Child Hospital (Madinah); King Abdulaziz Hospital and Oncology Center (Jeddah); Al-Nood Specialist Hospital (Makkah); King Abdulaziz Specialist Hospital (Taif); King Faisal Hospital (Taif); Arar Central Hospital (Arar); King Fahad Medical City; King Saudi Medical Complex (Riyadh); and King Khaled Hospital (Tabouk); Saudi Arabia | All included *P. aeruginosa* were R to IPM or MEM (MIC, ≥8 µg/ml) and CAZ (MIC, ≥32 µg/ml). Antimicrobial susceptibility screening for inclusion criteria was also performed by disc diffusion method as described by CLSI guidelines and susceptibility breakpoints (M100-S22). |
| **Khan & Faiz, 2016 [32]** | Clinical isolates from respiratory (87% lower tract and 13% upper tract), surgical, genital, urinary, blood, ear, eye and burn infections | August 2013–January 2014 | Saudi National Guard Hospital, Al-Noor Specialist Hospital, Maternity and Children Hospital, Maternity and Children Hospital, Saudi Arabia | An automated system was used for the identification and antibiotic susceptibility of isolates. Results were interpreted according to CLSI, 2015. |
| **Ibrahim, 2018 [33]** | Non-duplicate clinical specimens of ICU patients from blood, urine, sputum, tracheal aspirate, wound swab, eye swab, throat swab, umbilical discharge, ear swab, and high vaginal swab. Only specimens with complete patient information were included and isolates from the same specimens were excluded. | December 2016–January 2018 | King Abdullah Hospital, Saudi Arabia | The susceptibility test was carried out according to the recommendations of the CLSI (M07-A8 and M100-S25). Identified strains were tested against antimicrobial drugs using an automated microbiology system (PMID: 22909279). MDR isolates were R to >3 antimicrobial classes. |
| **Alhussain et al., 2021 [34]** | Non-duplicate clinical isolates from blood, urine, wound and sputum from hospital-acquired infections only. Patients with non-hospital-acquired infections, or with isolates from sources other than blood, urine, wound and sputum were excluded, or who had signs of infection at any other body site at the time of admission were excluded | January 2016–December 2018 | King Abdulaziz Medical City, Saudi Arabia | Antimicrobial susceptibility testing was performed using an automated system, according to the NCCLS (M100-S29). MDR was defined according to **[5]**. |
| **Bukhari et al., 2012 [35]** | Cultures of sputum, tracheal aspirate, pleural fluid, or blood from patients on a mechanical ventilator with ventilator-associated pneumonia. Non-ventilated patients in the ICU were excluded. | January–December 2010 | Hera General Hospital, Makkah, Saudi Arabia | Not reported |
| **Ayoub Moubareck et al., 2019 [36]** | Clinical isolates from drain fluid, wound, wound exudate, sputum, urine, broncho-alveolar lavage, respiratory fluid, blood, ear, foot ulcer, and unknown clinical specimens | June 2015–June 2016 | Dubai Hospital, Rashid Hospital, American Hospital, and Mediclinic City Hospital, UAE | All included *P. aeruginosa* isolates had reduced susceptibility to either IPM or MEM. Susceptibility testing to nine antimicrobial agents including imipenem, meropenem, piperacillin-tazobactam, ceftazidime, cefepime, amikacin, gentamicin, norfloxacin, ciprofloxacin, was done using the VITEK 2 Compact Analyzer. Susceptibility testing to colistin was done using broth microdilution. Results were interpreted according to CLSI 2017 criteria (M100-S27). MDR was defined according to **[5]**. |
| **Alatoom et al., 2017 [37]** | Bacteria were isolated from different clinical specimens including sputum, blood, urine, and other body tissues and fluids including wounds and abdomen. Only one isolate per patient infection episode was included in this study Bacterial isolates were not subdivided based on source of infection (i.e., intra-abdominal, urinary, pulmonary, etc).. | 2015–2016 | Cleveland Clinic Abu Dhabi, UAE | Isolates were tested for susceptibility to C/T and CZA using validated Etest strips. CLSI C/T breakpoints (S, ≤4 µg/ml; I, 8 µg/ml; R, ≥16 µg/ml) and FDA CZA breakpoints (S, ≤8 µg/ml and R, ≥16 µg/ml) for *P. aeruginosa* were used. Other drugs were tested by an automated susceptibility method according to CLSI criteria, except CST, for which EUCAST criteria were used for MIC interpretations (R, ≥4 µg/ml). MDR was defined according to **[5]**. |
| **Sid Ahmed et al., 2022 [38]** | Consecutive nonduplicate isolates collected from respiratory samples, skin and soft tissue, urine, blood, and other sample types. Most patients (56.8%) were colonized (*P. aeruginosa* from 2 consecutive cultures of samples from the same site with no evidence of infection) rather than infected. 56.% of patients were colonized. | October 2014–September 2017 | Hamad General Hospital, Rumaila Hospital, the Women’s Hospital, the Heart Hospital, and the National Centre for Cancer and Research, Doha, Qatar | The bacterial identification and initial antimicrobial susceptibility tests were performed using an automated identification and susceptibility testing system. MICs were determined using MIC test strips. The results were interpreted using CLSI reference breakpoints (M100-S30) MDR was defined according to **[5]** |
| **Al-Agamy et al., 2016 [39]** | Clinical isolates from hospitalized patients from samples (types not reported) | January–December 2011 | Single tertiary hospital, Saudi Arabia | Susceptibility testing was performed by an agar dilution method, and the data were interpreted according to the CLSI breakpoints (M100-S24). |
| **Abdalhamid et al., 2016 [40]** | Non-repetitive, nonduplicated rectal swab specimens colonized with *P. aeruginosa* from patients admitted to the ICU | February–May 2015 | King Fahad University Hospital and King Fahad Specialist Hospital, Saudi Arabia | Organism identification and antimicrobial susceptibility testing for these strains were carried out using an automatic system. MICs and breakpoints were determined according to the guidelines of CLSI (version not reported). |
| **Somily et al., 2021 [41]** | Non-duplicate clinical isolates from blood, urine, wound swabs, sputum, and other body sites and fluids. Adult and pediatric isolates were included with or without the presence of associated infection, and from healthcare-associated or community-associated infections. Duplicate isolates (defined as same person and same month but different body sites) were excluded. | 2011–2019 | King Saud University Medical City, King Abdulaziz Medical City, King Abdulaziz University Hospital Jeddah, King Fahad Medical City Riyadh, Prince Sultan Military Medical City, King Faisal Specialist Hospital and Research Center, and King Fahad University Hospital Dammam, Saudi Arabia | Susceptibility testing was performed by disk diffusion, following the guidelines of the CLSI (M100-S19). |
| **Al-Tawfiq et al., 2020 [42]** | The antibiogram included non-repetitive blood isolates (only one isolate per patient) | 2013–2018 | General hospital (name not given), Saudi Arabia | Antibacterial susceptibility tests and bacterial identification were done using an automated system. The interpretation of the antibacterial susceptibility tests was based on the CLSI guidelines (NCCLS M100-S11). |
| **Kazmierczak et al., 2016 [50]** | Nonduplicate, nonconsecutive isolates from intra-abdominal, urinary tract, skin and soft tissue, lower respiratory tract, and bloodstream infections | 2012–2014 | Asia-Pacific region, Europe, Latin America, the Middle East-Africa, and North America | Antibiotic susceptibility testing was performed by broth microdilution using custom frozen panels. Panel manufacture, inoculation, incubation, interpretation, and quality control testing were performed according to CLSI guidelines (M07-A9 and M100-S25). |

Abbreviations: AMK, amikacin; AZT, aztreonam; CAZ, ceftazidime; CF, cystic fibrosis; CIP, ciprofloxacin; CLSI, Clinical and Laboratory Standards Institute; CSF, cerebrospinal fluid; CST, colistin; CZA, ceftazidime-avibactam; ECV, epidemiological cutoff value; ETP ertapenem; EUCAST, European Committee on Antimicrobial Susceptibility Testing; FDA, The United States Food and Drug Administration; FEP, cefepime; I, intermediate; ICU, intensive care unit; IPM, imipenem; ISO, International Standards Organization; MBL, metallo-β-lactamase; MDR, multidrug-resistant or multidrug resistance; MEM, meropenem; MIC, minimum inhibitory concentration; NCCLS, National Committee for Clinical Laboratory Standards; NS, non-susceptible; R, resistant; S, susceptible; SMART, study for monitoring antimicrobial resistance trends; TZP, piperacillin-tazobactam; UAE, United Arab Emirates; and WHO, World Health Organization.

# Supplementary Table 3. Antimicrobial resistance or susceptibility data for *P. aeruginosa* from the Arabian Gulf countries and other countries/regions, stratified by testing method (2010–2021)

| **Study reference** | **Country / Region** | **Total *P. aeruginosa* (N)** | **%S or %R^a^ to antimicrobial agent** | | | | | | | | | | | | | | |
| --- | --- | --- | --- | --- | --- | --- | --- | --- | --- | --- | --- | --- | --- | --- | --- | --- | --- |
|  |  |  |  | **AMK** | **ATM** | **FEP** | **CAZ** | **CZA** | **C/T** | **CIP** | **CST** | **DOR** | **GEN** | **IPM** | **LVX** | **MEM** | **TZP** |
| **Broth microdilution method** | | | | | | | | | | | | | | | | | |
| **Kazmierczak et al., 2015 [8]** | Global / Middle East-Africa / Kuwait | 8,010 | %S | 90.2 | 61.4 | 78.6 | 77.4 | NT | NT | NT | 99.5 | NT | NT | NT | 71.7 | 73.3 | 69.1 |
| **Kiratisin et al., 2021 [9]** | Global / Middle East-Africa | 65 (CP) | %R | 95.4 | 32.3 | 96.9 | 100 | 92.3 | NT | NT | 0.0 | NT | NT | 96.9 | 100 | 95.4 | 98.5 |
| **Kadri et al., 2018 [10]** | United States | 4,493 | %R | NT | 17.3 (365) | 5.5 (652) | 7.0 (762) | NT | NT | 14.7 (860) | NT | 6.4 (78) | NT | 11.5 (494) | 16.7% (723) | 8.8 (514) | 5.8 (815) |
| **Karlowsky et al., 2021 [12]** | Middle East | 827 | %S | 92.7 | 67.8 | 81.0 | 80.5 | 92.4 | NT | NT | 99.5 | NT | NT | 66.0 | 62.8 | 72.9 | 74.8 |
|  |  | 315 (MDR) | %S | NT | NT | 50.2 | NT | 80.0 | NT | NT | NT | NT | NT | NT | NT | 40.4 | 35.6 |
|  |  | 61 (DTR) | %S | NT | NT | 0.0 | NT | 31.1 | NT | NT | NT | NT | NT | NT | NT | 0.0 | 0.0 |
| **Nichols et al., 2016 [13]** | Global / Middle East-Africa | 7,062 | %S | 89.4 | NT | 78.3 | 77.0 | 92.0 | NT | NT | 99.5 | 74.3 | NT | 61.4 | 71.9 | 72.7 | 68.6 |
|  |  | 689 | %S | 90.1 | NT | 79.5 | 80.8 | 91.7 | NT | NT | 99.6 | 77.1 | NT | 63.3 | 75.9 | 74.8 | 68.8 |
| **Karlowsky et al., 2018 [14]** | Global / Middle East | 12,170 | %S | 91.1 | 64.8 | 75.0 | 74.3 | NT | NT | 73.9 | 99.0 | NT | NT | 69.0 | 70.1 | NT | 69.1 |
|  |  | 3,708 (MDR) | %S | 72.9 | 10.6 | 21.1 | 20.3 | NT | NT | 36.5 | 97.9 | NT | NT | 28.8 | 31.5 | NT | 10.0 |
|  |  | 751 | %S | 89.4 | 65.3 | 76.8 | 75.6 | NT | NT | 77.0 | 99.6 | NT | NT | 63.3 | 72.0 | NT | 69.5 |
| **Moise et al., 2021 [15]** | Global / Middle East | 1,783 | %S | NT | NT | NT | 66.0 | NT | 87.1 | NT | NT | NT | NT | NT | NT | 61.6 | 62.4 |
|  |  | 684 (MEM-NS) | %S | NT | NT | NT | 34.9 | NT | 68.1 | NT | NT | NT | NT | NT | NT | 0.0 | 28.2 |
| **Broth microdilution method and automated system** | | | | | | | | | | | | | | | | | |
| **Ayoub Moubareck et al., 2019 [36]** | United Arab Emirates | 37 (carbapenem-NS) | % R | 32.4 | NT | 32.4 | 35.1 | NT | NT | 43.2 | NT | NT | 35.1 | 97.3 | NT | 64.9 | 43.2 |
| **Broth microdilution method and disk diffusion method** | | | | | | | | | | | | | | | | | |
| **Alfouzan et al., 2018 [18]** | Kuwait | 48 (MDR) | %S | 31.2 | 47.9 | 25.0 | 22.9 | 39.6 | 33.3 | 16.7 | 97.9 | NT | NT | 10.4 | NT | 8.3 | 14.6 |
| **Disk diffusion method** | | | | | | | | | | | | | | | | | |
| **Joji et al., 2019 [16]** | Bahrain | 50 (CR) | %R | 72.0 | NT | NT | 86.0 | NT | NT | 100 | 0.0 | NT | 86.0 | 80.0 | NT | 90.0 | 90.0 |
| **Al Rashed et al., 2020 [17]** | Bahrain | 50 (CIP‑R) | %R | 72.0 | NT | NT | 86.0 | NT | NT | 100 | 0.0 | NT | 86.0 | 88.0 | NT | 90.0 | 90.0 (piperacillin) |
| **Alhubail et al., 2020 [19]** | Kuwait | 102 | %S | 87.0 | NT | NT | 65.7 | NT | NT | 77.2 | NT | NT | 80.0 | 85.0 | NT | 88.4 | 71.1 |
| **Al Rahmany et al., 2019 [20]** | Oman | 2,362 | %S | 80.0 | NT | NT | 90.0 | NT | NT | 85.0 | 100.0 | NT | 84.0 | NT | NT | 58.0 | 93.0 |
| **Somily et al., 2021 [41]** | Saudi Arabia | 73,728 | Change (+/-) in %R | 16.3−10.4; change: -5.9% | 25.8–43.0; change, +17.3% | 21.3–21.3; change: 0.0% | 22.2–24.9; change: +2.7% | NT | NT | 18.3–22.6; change: +4.3% | 2.0–1.9; change: -0.1% | NT | 15.7–14.7; change: -1.0% | 24.9–37.1; change: +12.3% | 22.0–24.6 change: +2.6% | 21.9–33.5; change: +11.6% | 27.2–30.9 change: +3.6% |
| **E-test method** | | | | | | | | | | | | | | | | | |
| **Somily et al., 2012 [30]** | Saudi Arabia | 33 (MDR) | %R | NT | NT | NT | NT | NT | NT | NT | 6.1 | NT | NT | 90.9 | NT | 81.8 | NT |
| **Disk diffusion method and E-test method** | | | | | | | | | | | | | | | | | |
| **Tawfik et al., 2012 [28]** | Saudi Arabia | 156 | %R | 26.3 | 16.7 | 20.5 | 22.4 | NT | NT | 30.1 | NT | NT | 40.8 | 16.2 | NT | NT | 21.8 |
|  | Saudi Arabia | 35 (CAZ-R) | %R | 82.5 | 48.6 | 88.6 | NT | NT | NT | 90.0 | NT | NT | 87.5 | 70.0 | NT | NT | 71.1 |
| **Al-Agamy et al., 2012 [29]** | Saudi Arabia | 200 | %R | 26.5 | 16.5 | 20.5 | 19.5 | NT | NT | 30.0 | NT | NT | 41.0 | 16.0 | NT | NT | 22.5 |
| **Automated system** | | | | | | | | | | | | | | | | | |
| **Khan & Faiz, 2016 [32]** | Saudi Arabia | 121 | %R | 7.4 | 16.5 | 8.3 | 14.0 | NT | NT | 16.5 | NT | NT | 11.6 | 19.0 | NT | 30.6 | 4.9 |
| **Ibrahim, 2018 [33]** | Saudi Arabia | 69 | %R | 12/64 (18.8) | 34/64 (53.1) | 32/60 (53.3) | 28/67 (41.8) | NT | NT | 24/64 (37.5) | 3/10 (30.0) | NT | 29/63 (31.7) | 26/68 (38.2) | NT | 32/61 (52.5) | 31/67 (46.3) |
| **Alhussain et al., 2021 [34]** | Saudi Arabia | 90 | %S | 86.7 | NT | 84.4 | 77.8 | NT | NT | 71.1 | NT | NT | 86.7 | 58.9 | NT | 58.9 | 71.1 |
| **Al-Tawfiq et al., 2020 [42]** | Saudi Arabia | 4,210 | %S (study year) | 91.1 (2017, 2018)–97.9 (2013, 2016) | NT | 80.0 (2017)–91.0 (2013) | 80.0 (2017)–92.1 (2013) | NT | NT | 71.9 (2015)–86.1 (2013) | NT | NT | 86.2 (2015)–93.0 (2014) | 69.9 (2015)–82.1 (2014, 2018) | NT | 67.1 (2015)–89.1 (2014) | 85.1 (2017, 2018)–93.9 (2013) |
| **Automated system and E-test method** | | | | | | | | | | | | | | | | | |
| **AbdulWahab et al., 2017 [23]** | Qatar | 61 | %S | 67.2 | NT | 70.5 | NT | NT | NT | 77.0 | NT | NT | 59.0 | NT | NT | 88.5 | 90.2 |
|  |  | 12 (MDR) | %R | 100 | NT | 100 | NT | NT | NT | 91.7 | NT | NT | 100 | NT | NT | 58.3 | 50.0 |
| **Alatoom et al., 2017 [37]** | United Arab Emirates | 31 | %R or %S | NT | NT | 19.4 | 25.8 | 93.5 (%S) | 96.8 (%S) | NT | NT | NT | 16.1 | NT | NT | 51.6 | 35.5 |
| **Automated system and MIC test strips** | | | | | | | | | | | | | | | | | |
| **Sid Ahmed et al., 2019a [24]** | Qatar | 205 (MDR) | %R | 58.0 | NT | 96.6 | NT | NT | NT | 91.2 | 3.4 | NT | 73.2 | NT | NT | 90.2 | 90.2 |
| **Sid Ahmed et al., 2019b [26]** | Qatar | 205 (MDR) | %R | NT | NT | NT | NT | 68.8 | 62.9 | NT | NT | NT | NT | NT | NT | NT | NT |
| **Sid Ahmed et al., 2020b [27]** | Qatar | 75 (MDR) | %S | NT | 21.3 | 4.0 | 28.0 | 48.0 | 40.0 | NT | NT | NT | NT | NT | NT | 9.3 | 10.7 |

Abbreviations: AMK, amikacin; ATM, aztreonam; CAZ, ceftazidime; CIP, ciprofloxacin; CP, carbapenemase-producing; CR, carbapenem-resistant; CST, colistin; C/T, ceftolozane-tazobactam; CZA, ceftazidime-avibactam; DOR, doripenem; DTR, difficult-to-treat resistance; FEP, cefepime; GEN, gentamicin; IPM, imipenem; LVX, levofloxacin; MBL, metallo-β-lactamase; MDR, multidrug-resistant; MEM, meropenem; MIC, minimum inhibitory concentration; NS, non-susceptible; NT, not tested; R, resistant; S, susceptible; TZP, piperacillin-tazobactam; and UAE, United Arab Emirates.

^a^Numerators and denomators are given for an antimicrobial agent if the tested denominator values differed from value shown in the total isolate column.

# Supplementary Table 4. Distribution of β-lactamase genes among *P. aeruginosa* from the Arabian Gulf countries and other countries/regions (2010–2021)

| **Region/Country** | **IMP (n [%])** | **NDM (n [%])** | **VIM (n [%])** | **GES (n [%])** | **KPC (n [%])** |
| --- | --- | --- | --- | --- | --- |
| Arabian Gulf/ Bahrain **[16]** | not tested | NDM-type (1 [2.5]) | VIM-type, VIM-2 and VIM-4 (19 [47.5]) | not tested | not tested |
| Arabian Gulf/ Kuwait **[8]** | IMP-type (0) | NDM-type (0) | VIM-2 (11) and VIM-4 (3) | not tested | KPC-type (0) |
| Arabian Gulf/ Qatar **[26, 27]** | IMP-1 (3 [3.5]) | NDM-type (0) | VIM-2 (20) and VIM-5 (2); (22 [25.9]) | GES-type (0) | KPC-type (0) |
| Arabian Gulf/ Saudi Arabia **[8, 28, 29, 31, 40]** | IMP-7 (10 [7.6]) | NDM-type (4 [3.0]) | VIM-type, VIM-1, VIM-2, VIM-4, VIM-6, VIM-11 and VIM-28 (50 [37.9]) | GES-type, GES-1, GES-4, GES-5 and GES-6 (16 [12.1]) | KPC-type (0) |
| Arabian Gulf/ UAE **[36]** | IMP-type (0) | NDM-type (0) | VIM-type, VIM-2, VIM-30, VIM-31 and VIM-42 (12 [32.4]) | GES-9 and GES-5 (2 [5.4]) | KPC-type (0) |
| Asia/ India **[43–46]** | IMP-type (14 [6.1]) | NDM-type (137 [59.3]) | VIM-type (135 [58.4]) | GES-type (13 [8.3]) | KPC-type (0) |
| Asia/ Pakistan **[47–49]** | IMP-type (117 [34.5]) | NDM-type (50 [14.7]) | VIM-type (132 [38.9]) | GES-type (24 [100]) | KPC- type (39 [15.5]) |
| EMR incl Greece/ Greece, Turkey and Israel* **[8, 50, 51]** | IMP-type (0) | NDM-type (0) | VIM-type (65), VIM-2 (24), VIM-4 (3), and VIM-5 (1); (93 []) | GES-1 (1) | KPC-type (0) |
| Southern Europe excl Greece/ Italy, Portugal, and Spain **[8, 50, 51]** | IMP-type (0) | NDM-type (2) | VIM-type (38), VIM-1 (5), VIM-2 (6) and VIM-44 (1); (50) | GES-type (52) and GES-1 (5) | KPC-type (0) |
| North America/ United States and Canada **[8, 50, 52]** | IMP-1 (2), IMP-7 (2), IMP-13 (2) and IMP-18 (2); (8) | NDM-type (0) | VIM-2 (2) | GES-5 (7) | KPC-type (0) |
| Latin America/ Argentina, Brazil, Chile, Colombia, Mexico, and Venezuela **[8, 9, 50]** | IMP-type (26), IMP-1 (1), IMP-16 (1), IMP-18 (2), and IMP-49 (1) | NDM-type (0) | VIM-type (153), VIM-2 (52) | GES-type (33) | KPC-type (54) and KPC-2 (28) |
| Southeast Asia/ Malaysia, Philippines, Singapore, and Thailand **[8, 46, 50]** | IMP-type (30), IMP-1 (4), IMP-7 (1), IMP-14 (1), IMP-26 (5) and IMP-48 (6) | NDM-type (12) | VIM-type (53), VIM-2 (25), VIM-5 (2), VIM-6 (1) and VIM-45 (2) | GES-type (1) | KPC-type (0) |

Abbreviations: EMR, Eastern Mediterranean Region; inc, including; excl, excluding; NDM, New Delhi metallo-β-lactamase; VIM, Verona integron-encoded metallo-β-lactamase; IMP, imipenemase; GES, Guiana extended-spectrum; KPC, Klebsiella pneumoniae carbapenemase; and UAE, United Arab Emirates.
